# Lignan Glycosides from *Urena lobata*

**DOI:** 10.3390/molecules24152850

**Published:** 2019-08-06

**Authors:** Yuan Luo, Cong Su, Ning Ding, Bowen Qi, Fangfang Jia, Xiping Xu, Xiao Liu, Juan Wang, Xiaohui Wang, Pengfei Tu, Shepo Shi

**Affiliations:** 1 Modern Research Center for Traditional Chinese Medicine, School of Chinese Materia Medica, Beijing University of Chinese Medicine, Beijing 100029, China; 2 Beijing Key Lab for Quality Evaluation of Chinese Meteria Medica, Beijing University of Chinese Medicine, Beijing 100029, China

**Keywords:** *Urena lobata* L., lignan glycosides, urenalignosides A–D, nitric oxide production

## Abstract

Four new lignan glycosides; urenalignosides A–D (**1**–**4**), along with 12 known ones (**5**–**16**) were isolated from *Urena lobata*. Their structures were determined on the basis of extensive spectroscopic and spectrometric data (1D and 2D NMR; IR; CD; and HRESIMS). Compounds **2**–**4**; **6**; **7**; **10**; and **11** showed inhibition of nitric oxide production in lipopolysaccharide-induced RAW 264.7 macrophage cells with IC_50_ values in the range of 25.5–98.4 μM (positive control; quercetin; IC_50_ = 7.2 ± 0.2 μM).

## 1. Introduction

**Figure 1:**
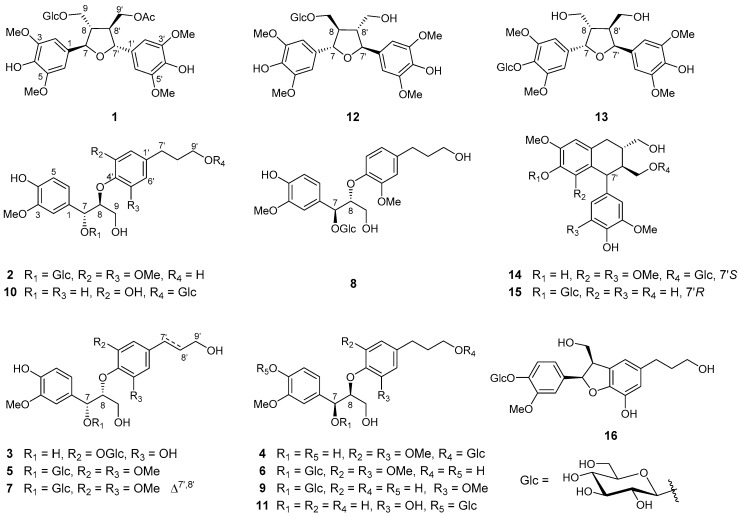
Structures of compounds **1**–**16** from *U. lobate*.

*Urena lobata*, belonging to the family Malvaceae, is an annually shrubby herbage widely distributed around the world, particularly in the tropical and subtropical areas of Asia, South America, and Africa [1]. This plant is also known as Caesar weed, Congo jute, and Bachita, the local name varies from region to region. In Africa, the leaves and flowers of *U. lobata* could be eaten as food during famine time and the bast fiber of *U. lobata* is used as cordage material [2]. More interestingly, *U. lobata* is also commonly used in folk medicines for the treatment of diabetes, abdominal colic, malaria, gonorrhea, dysentery, fever, rheumatism, and edema [3,4]. Pharmacological studies indicated that the extract of *U. lobata* showed significant antibacterial, antihyperglycemic, antinociceptive, antidiarrheal, anti-inflammatory, and wound healing activities [5,6,7]. In China, *U. lobata* is also named “Ditaohua,” which is dominantly distributed in the south of China, such as Guangxi, Yunnan, and Guizhou provinces and clinically used to treat pathological leucorrhea and gonorrhea [8]. Promoted by these significant activities, great efforts have been made to clarify the bioactive constituents of *U. lobata* leading to the separation and elucidation of flavonoids, phenylethyl glycosides, lignans, coumarins, and triglycerides [1,9,10,11,12,13]. In our previous report, 16 megastigmane glycosides were identified from *U. lobata* [14]. As an ongoing study, four new lignan glycosides, urenalignosides A–D (**1**–**4**) together with 12 known ones (**5**–**16**) were obtained from *U. lobata* (Figure 1). Herein, the isolation and structural elucidation of the new compounds, as well as their inhibitory effects on NO production on LPS-stimulated RAW264.7 macrophage cells, are described.

## 2. Results

The 95% EtOH extracts of *U. lobata* were suspended in H_2_O and extracted successively with petroleum ether (PE), EtOAc, and *n*-BuOH. The *n*-BuOH soluble fraction was separated by D_101_ macroporous adsorption resin, silica gel, and Sephadex LH-20 column chromatography and semi-preparative HPLC to afford four new lignan glycosides (**1**–**4**) together with 12 known ones (**5**–**16**) (Figure 1).

**Figure 2:**
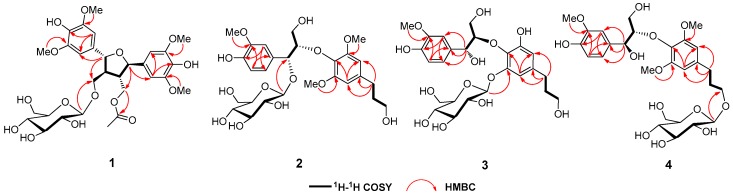
Key HMBC and ^1^H-^1^H COSY correlations of compounds **1**–**4**.

Compound **1** was obtained as a colorless powder. Its molecular formula was assigned as C_30_H_40_O_15_ due to the presence of a [M − H] ^−^ ion at *m/z* 639.2282 (calcd for C_30_H_39_O_15_, 639.2294) in the HRESIMS spectrum (Figure S1), which was also supported by the ^13^C-NMR data (Table 1). The IR spectrum of **1** showed the absorption bands contributing to hydroxy group (3385 cm^−1^), benzene ring (1615 and 1518 cm^−1^), and ester carbonyl (1735 cm^−1^) group. The NMR spectra of **1** (Figures S2 and S3) showed the presence of two 1,3,4,5-tetrasubstituted benzene moieties [*δ*_H_ 6.78 (2H, s, H-2,6), 6.80 (2H, s, H-2′,6′). *δ*_C_ 134.1 (C-1), 105.2 (C-2,6), 149.4 (C-3,5), 136.3 (C-4); 133.6 (C-1′), 104.8 (C-2′,6′), 149.3 (C-3′,5′), 136.4 (C-4′)], two oxygenated methines [*δ*_H_ 5.12 (1H, d, *J* = 8.0 Hz, H-7), 4.98 (1H, d, *J* = 9.0 Hz, H-7′). *δ*_C_ 85.6 (C-7), 84.2 (C-7′)], two *sp*^3^ methines [*δ*_H_ 2.75 (1H, m, H-8), 2.45 (1H, m, H-8′). *δ*_C_ 52.7 (C-8), 51.1 (C-8′)], two oxygenated methylenes [*δ*_H_ 4.36 (1H, overlapped, H-9′a) and 3.72 (1H, dd, *J* = 12.0, 5.0 Hz, H-9′b); 4.09 (1H, dd, *J* = 10.0, 5.5 Hz, H-9a) and 3.80 (1H, dd, *J* = 10.0, 4.5 Hz, H-9b). *δ*_C_ 69.3 (C-9), 64.8 (C-9′)], and four methoxyl groups [*δ*_H_ 3.93 (12H, s), *δ*_C_ 56.9]. Comparison of the above NMR data with those of icariol A_2_ [15], a lignan previously isolated from *Epimedium sagittatum*, revealing the presence of an icariol A_2_ moiety in **1**. In addition, signals due to an acetyl group [*δ*_H_ 1.95 (3H, s), *δ*_C_ 20.7, 172.8] and a glucopyranosyl moiety were also observed in the NMR spectra of **1**. The anomeric proton was presented at *δ*_H_ 4.36 (1H, d, *J* = 8.0 Hz), corresponding to the carbon at *δ*_C_ 104.6 assigned by HSQC experiment, and the relatively large coupling constant (*J* = 8.0 Hz) of the anomeric proton suggested that the glucopyranosyl moiety was in *β* configuration. Given that naturally occurring glucose is D-form, and limited by the small amount of **1**, we tentatively determined the glucopranosyl moiet in **1** was in D-form. In the HMBC spectra of **1**, the correlations between the anomeric proton *δ*_H_ 4.36 (1H, d, *J* = 8.0 Hz, H-1′′) and C-9 (*δ*_C_ 69.3) confirmed that the glucopyranosyl moiety was linked at C-9 (Figure 2). The acetyl group was linked at C-9′ determined by the HMBC correlation between H-9′ and the carbonyl carbon (*δ*_C_ 172.8). All the protons and carbons were unambiguously assigned (Table 1) by ^1^H-^1^H COSY, HSQC, and HMBC experiments (Figures S4–S6).

The relative configuration of **1** was determined by NOESY spectrum (Figure S7), which showed the NOE correlations of H-7/H-8′ and H-7′/H-8. The CD spectra (Figure S8) of **1** showed the positive Cotton effect at 246 nm suggested that both C-7 and C-7’ were in *R* configuration [16,17], and thus the configuration of C-8, and C-8’ were assigned as 8*S*, 8’*S*. Accordingly, the structure of **1** was determined as shown in Figure 1, named as *urenalignoside A*.

Compound **2** was obtained as colorless powder. Its molecular formula was assigned as C_27_H_38_O_13_ by the [M + HCOO]^–^ ion at *m/z* 615.2284 (calcd for C_28_H_39_O_15,_ *m/z* 615.2294) in the HRESIMS spectrum (Figure S9), which was also supported by the ^13^C-NMR -NMR data (Table 1). The NMR spectra of **2** (Figures S10 and S11) showed the presence of a 1,3,4-trisubstituted [*δ*_H_ 7.28 (1H, d, *J* = 1.5 Hz, H-2), 6.83 (1H, d, *J* = 8.0 Hz, H-5), 6.96 (1H, dd, *J* = 8.0, 1.5 Hz, H-6). *δ*_C_ 130.8 (C-1), 113.3 (C-2), 148.7 (C-3), 147.1 (C-4), 115.5 (C-5), 122.1 (C-6)] and a 1,3,4,5-tetrasubstituted benzene moieties [*δ*_H_ 6.53 (2H, s, H-2′,6′). *δ*_C_ 135.1 (C-1′), 106.7 (C-2′,6′), 154.3 (C-3′,5′), 139.9 (C-4′)], two oxygen-bearing methines [*δ*_H_ 5.31 (1H, d, *J* = 3.0 Hz, H-7), 4.23 (1H, m, H-8). *δ*_C_ 77.7 (C-7), 86.8 (C-8)], two oxygen-bearing methylenes [*δ*_H_ 3.61 (2H, t, *J* = 6.4 Hz, H-9′), 3.16 (2H, m, H-9). *δ*_C_ 62.8 (C-9′), 61.4 (C-9)], two methylenes [*δ*_H_ 2.67 (2H, t, *J* = 7.5 Hz, H-7′), 1.86 (2H, m, H-8′). *δ*_C_ 33.4 (C-7′), 35.4 (C-8′)], and three methoxy groups [*δ*_H_ 3.89 (3H, s, 3-OCH_3_), 3.74 (6H, s, 3′,5′-OCH_3_). *δ*_C_ 56.4 (3, 3′, 5′-OCH_3_)]. Comparison of the above-mentioned NMR data with those of 1-(4′-hydroxy-3′-methoxy-phenyl)-2-[4′′-(3-hydroxypropyl)-2′′,6′′-dimethoxyphenoxy] propane-1,3-diol, a lignan previously isolated from *Bursera tonkinensis* [18], suggested the occurrence of an 8-*O*-4′-neolignan moiety in **2**. In addition, signals due to a glucopyranosyl moiety were also observed in the NMR spectra of **2**. The relatively large coupling constant (*J* = 7.5 Hz) of the anomeric proton resonated at *δ*_H_ 4.23 (1H, d, *J* = 7.5 Hz, H-1′′) suggested the glucopyranosyl moiety was in *β* configuration. The linkage of the glucopyranosyl moiety was determined at C-7 by the HMBC correlation between the anomeric proton and C-7 (Figure 2). Unambiguous assignments of the protons and carbons (Table 1) were achieved by ^1^H-^1^H COSY, HSQC, HMBC, and NOESY experiments (Figures S12–S15).

It has been well reported that the relative configurations of C-7 and C-8 could be solved by the analysis of the coupling constant between H-7 and H-8. Regularly, a relatively small coupling constant (*J* = 3–4 Hz) between H-7 and H-8 defines the *erythro* configurations of C-7 and C-8, while a relatively large coupling constant (*J* = 6–8 Hz) give rise to the *threo* configurations of C-7 and C-8 [19,20,21,22,23,24]. Accordingly, the stereochemistry of C-7 and C-8 in **2** were assigned as *erythro* according to the small coupling constant (*J* = 3.0 Hz) between H-7 and H-8. The positive Cotton effect at 233 nm in the CD spectrum (Figure S16) of **2** suggested that the configuration of C-8 was *S* [22,24,25,26], and thus the configuration of C-7 was determined as *R*. Therefore, the structure of **2** namely *urenalignoside B* was elucidated as shown in Figure 1. 

Compound **3** was obtained as a colorless powder, with a molecular formula of C_25_H_34_O_13_ determined by the presence of a [M − H]^–^ ion at *m/z* 541.1920 (calcd for C_25_H_33_O_13_, *m/z* 541.1927) in the HRESIMS spectrum (Figure S17). The NMR data of **3** (Figures S18–S23) is comparable to those of **2**, except the absence of one methoxy group in **3**. In the HMBC spectrum of **3**, the correlation between the anomeric proton [*δ*_H_ 4.93 (1H, *J* = 7.5 Hz, H-1′′)] of the glucopyranosyl moiety and the C-3’ of the aglycon demonstrated that the glucopyranosyl moiety was linked at C-3’ in **3** (Figure 2). The large coupling constant (*J* = 8.5 Hz) between H-7 and H-8 suggested that the C-7 and C-8 were in *threo* orientation. The negative Cotton effect at 233 nm in the CD spectrum (Figure S24) of **3** suggested that the configuration of C-8 was *R* [23,24], and thus the configuration of C-7 was 7*R*. Therefore, the structure of **3** namely *urenalignoside C* was determined as shown in Figure 1.

Compound **4** was obtained as a colorless powder, with a molecular formula of C_27_H_38_O_13_ by the [M − H]^–^ ion *m/z* 569.2258 (calcd for C_27_H_37_O_13_, *m/z* 569.2240) in the HRESIMS spectrum (Figure S25). Comparison of the NMR data of **4** (Figures S26–S31) with those of **2** revealed that these two compounds share a highly similar skeleton, except the significantly deshielded chemical shift of C-9′ (*δ*_C_ 69.2; ∆*δ*_C_ + 6.3), suggesting that the *O*-glucopyranosyl moiety was linked at C-9′ in **4**, but not like that at C-7 in **2**. The deduction was confirmed by HMBC correlation between the anomeric proton [*δ*_H_ 4.32 (1H, *J* = 8.0 Hz, H-1′′)] and C-9′ (Figure 2). The relatively large coupling constant (*J* = 7.0 Hz) between H-7 and H-8 suggested that the C-7 and C-8 were in *threo* orientation. The absolute configuration of C-8 was assigned as *S* based on the positive Cotton effect at 233 nm presented in the CD spectrum (Figure S32) of **4** [22,24,25,26], and thus the configuration of C-7 was assigned as *S*. Accordingly, the structure of **4** namely *urenalignoside D* was determined as shown in Figure 1.

By comparison of their spectroscopic and specific rotation data with those of the known compounds, the remaining 11 compounds were identified as (7*R*,8*R*)-*threo*-4,9,9’-trihydroxy-3,3’,5’-trimethoxy-8-*O*-4’-neolignan-7-*O*-*β*-d-glucopyranoside (**5**) [21], rourinoside (**6**) [22], (7*R*,8*R*)-*threo*-guaiacylglycerol-8-*O*-4’-sinapyl ether-7-*O*-*β*-d-glucopyranoside (**7**) [23], (7*S*,8*R*)-*erythro*-4,9,9’-trihydroxy-3,3’-dimethoxy-8-*O*-4’-neolignan-7-*O*-*β*-d-glucopyranoside (**8**) [24], (7*S*,8*S*)-*threo*-4,9,9’-trihydroxy-3,3’-dimethoxy-8-*O*-4’-neolignan-7-*O*-*β*-d-glucopyranoside (**9**) [24], (–)-(7*R*,8*S*)-4,7,9,3′,9′-pentahydroxy-3-methoxy-8-*O*-4′-neolignan-9′-*O*-*β*-d-glucopyranoside (**10**) [25], (7*S*,8*S*)-4,7,9,3’,9’-pentahydroxy-3-methoxyl-8-*O*-4’-neolignan-4-*O*-*β*-d-glucopyranoside (**11**) [26], (7*S*,7’*S*,8*R*,8’*R*)-icariol A_2_-9-*O*-*β*-d-glucopyranoside (**12**) [16], (7*S*,7’*S*,8*S*,8’*S*)-icariol A_2_-4-*O*-*β*-d-glucopyranoside (**13**) [27], lyoniresinol-9’-*O*-*β*-d-glucopyranoside (**14**) [28], (−)-isolariciresinol 4-*O*-*β*-d-glucopyranoside (**15**) [29], and cedrusin-4’-*O*-*β*-d-glucopy ranoside (**16**) [30], respectively. Compounds **2**–**11** and **16** are neolignans which are classified as a subgroup of lignan family [31].

## 3. Materials and Methods 

### 3.1. General Experimental Procedures

Optical rotations were obtained on a Rudolph Autopol IV automatic polarimeter (Hackettstown, NJ, USA). IR spectra were recorded on a Thermo Nicolet Nexus 470 FT-IR spectrophotometer (Madison, WI, USA) with KBr pellets. UV spectra were obtained using a Shimadzu UV-2450 spectrophotometer (Tokyo, Japan). NMR spectra were recorded on a Varian INOVA-500 spectrometer (Palo Alto, CA, USA) operating at 500 MHz for ^1^H-NMR and 125 MHz for ^13^C-NMR. HRESIMS was recorded on an LCMS-IT-TOF system, fitted with a Prominence UFLC system and an ESI interface (Shimadzu, Kyoto, Japan). Silica gel (200–300 mesh, Qingdao Marine Chemical Inc., Qingdao, China), LiChroprep RP-C_18_ gel (40–63 μm, Merck, Germany), D_101_ m acroporous adsorption resin (Qingdao Marine Chemical Inc., Qingdao, China) and Sephadex LH-20 (Qingdao Marine Chemical Inc., Qingdao, China) were used for open column chromatography (CC). HPLC was performed on a ShimadzuLC-20AT pump system (Shimadzu Corporation, Tokyo, Japan), equipped with an SPD-M20A photodiode array detector monitoring at 254 nm. A semi-preparative HPLC column (YMC-Pack C_18_, 250 × 10 mm, 5 μm) was employed for the isolation. TLC was performed using GF_254_ plates (Qingdao Marine Chemical Inc., Qingdao, China).

### 3.2. Plant Material

*Urena lobata* L. was collected in Guangxi Province, People’s Republic of China, in September 2013. The plant material was authenticated by one of the authors (P.F. Tu) and a voucher specimen (DTH2013029) was deposited at the Modern Research Center for Traditional Chinese Medicine, Beijing University of Chinese Medicine, Beijing, China.

### 3.3. Extraction and Isolation

The air-dried *U. lobata* (13.6 kg) were refluxed with 95% EtOH for three times (3 × 180 L, each for 1 h). After removing the solvent under reduced pressure, the residue (1.35 kg) was suspended in water (6 L), and partitioned with petroleum ether (3 × 6 L), EtOAc (5 × 6 L), and *n*-BuOH (3 × 6 L), successively. The *n*-BuOH-soluble fraction (158 g) was subjected to D_101_ macroporous adsorption resin column and eluted with H_2_O–EtOH (100:0, 90:10, 50:50, 20:80, 0:100) to yield five fractions (Fr. 1-5). Fr. 2 (20 g) and Fr. 3 (40 g) were combined and subjected to silica gel chromatography and eluted with a stepwise gradient of EtOAc-MeOH-H_2_O from 30:2:1 to 5:2:1 to give five subfractions (Subfr. A–E). Subfr. B (8 g) was chromatographed on a Sephadex LH-20 column and eluted with MeOH to give six subfractions (Subfr. B1–B6). Subfr. B3 (1 g) was chromatographed on a silica gel column and eluted with gradient of CH_2_Cl_2_–MeOH (12:1, 10:1, 8:1, 5:1, 1:1, v/v) to give seven subfractions (Subfr. B3a–B3g). Subfr. B3d (0.2 g) was purified by semipreparative HPLC using 27% aqueous MeCN as the mobile phase to afford compound **7** (2.1 mg, *t*_R_ 34.5 min). Subfr. B3g (0.1 g) was applied to semi-preparative HPLC using 25% aqueous MeCN to obtain two compounds **8** (3.1 mg, *t*_R_ 23.0 min) and **9** (4.2 mg, *t*_R_ 48.5 min). Subfr. B4 (4 g) was subjected to RP-C_18_ open column and eluted with a stepwise gradient of MeOH–H_2_O (1:4, 1:3, 1:2, 2:3, 1:0, v/v), to afford five fractions (Subfr. B4a–Subfr. B4e). Subfr. B4a (1.2 g) was applied to semi-preparative HPLC using 25% aqueous MeCN to give compound **1** (1.2 mg, *t*_R_ 28.5 min). Subfr. B4c (1.1 g) was further separated by ODS column chromatography and eluted with MeOH–H_2_O (1:19→1:3) to obtain six fractions (Subfr. B4c1–B4c6). Subfr. B4c4 was repeatedly separated and purified by semi-preparative HPLC (27% aqueous MeCN) to give two fractions Subfr. B4c4-1 (25.3 mg, *t*_R_ 40.0 min), Subfr. B4c4-2 (7.4 mg, *t*_R_ 49.0 min), and five compounds **3** (3.0 mg, *t*_R_ 44.5 min), **4** (2.1 mg, *t*_R_ 30.0 min), **5** (2.5 mg, *t*_R_ 36.0 min), **12** (7.5 mg, *t*_R_ 23.5 min), and **13** (2.5 mg, *t*_R_ 27.5 min). Subfr. B4c4-2 was purified by semi-preparative HPLC (30% aqueous MeOH) to give compounds **10** (1.8 mg, *t*_R_ 55.5 min) and **11** (2.0 mg, *t*_R_ 57.0 min). Subfr. B4c5 was applied to semi-preparative HPLC using 10% aqueous MeOH to give compounds **2** (2.5 mg, *t*_R_ 32.0 min), **6** (3.2 mg, *t*_R_ 37.0 min), **14** (2.1 mg, *t*_R_ 43.5 min), **15** (1.8 mg, *t*_R_ 54.0 min), and **16** (1.2 mg, *t*_R_ 55.5 min).

*Urenalignoside A* (**1**): Colorless powder, ${\left[\alpha \right]}_{D}^{25}$: −45.7 (*c* 0.1, MeOH); UV *λ* (log *ε*): 208 (4.49), 317 (4.31), 383 (3.91) nm; IR (KBr) ν_max_: 3385, 2921, 1735, 1615, 1518, 1462, 1428, 1367, 1331, 1217, 1114, 1076, 1036 cm^−1^; ^1^H and ^13^C-NMR data (see Table 1); negative-ion HRESIMS: *m*/*z* 639.2282 [M – H]^–^ (calcd for C_30_H_39_O_15_, 639.2294).

*Urenalignoside B* (**2**): Colorless powder, ${\left[\alpha \right]}_{D}^{25}$: −64.0 (*c* 0.1, MeOH); UV *λ* (log *ε*): 202 (4.14),226 (4.25), 277 (3.37), 298 (2.63), 317 (2.48), 329 (2.40), 341 (2.43), 348 (2.38) nm; IR (KBr) ν_max_: 3423, 2926, 1630, 1384, 1253, 1119, 1076, 1037 cm^−1^; ^1^H and ^13^C-NMR data (see Table 1); negative-ion HRESIMS: *m*/*z* 615.2284 [M + HCOO]^−^ (calcd for C_28_H_39_O_15_, 615.2294).

*Urenalignoside C* (**3**): Colorless powder, ${\left[\alpha \right]}_{D}^{25}$: −52.4 (*c* 0.1, MeOH); UV *λ* (log *ε*): 212 (4.58), 285 (4.00) nm; IR (KBr) ν_max_: 3389, 2968, 2923, 2852, 1739, 1610, 1456, 1431, 1366, 1259, 1228, 1216, 1174, 1111, 1028 cm^−1^; ^1^H and ^13^C-NMR data (see Table 1); negative-ion HRESIMS: *m*/*z* 541.1920 [M − H]^−^ (calcd for C_25_H_33_O_13_, 541.1927).

*Urenalignoside D (***4***)*: Colorless powder, ${\left[\alpha \right]}_{D}^{25}$: −54.0 (*c* 0.1, MeOH); UV *λ* (log *ε*): 207 (4.62), 263 (4.70), 316 (4.23) nm; IR (KBr) ν_max_: 3739, 3716, 3660, 3430, 2956, 2924, 2853, 1717, 1592, 1514, 1488, 1455, 1428, 1383, 1367, 1230, 1157, 1125, 1023 cm^−1^; ^1^H and ^13^C-NMR data (see Table 1); negative-ion HRESIMS: *m*/*z* 569.2258 [M − H]^−^ (calcd for C_27_H_37_O_13_, 569.2240).

### 3.4. Biological Assays

The murine macrophage RAW264.7 cell line was purchased from Peking Union Medical College (PUMC) Cell bank (Beijing, China), and was cultured in DMEM supplemented with 10% Fetal Bovine Serum, 100U/mL penicillin G and 100 μg/mL streptomycin, in a humidified 5% CO_2_ at 37 °C. Cell viability was evaluated using MTT assay. The NO concentration was detected by the Griess method. Briefly, RAW264.7 macrophage cells were seeded into 96-well plates at a density of 5 × 10^4^ cells/well and stimulated with 0.5 μg/mL LPS (Sigma, St. Louis, MO, USA) in the presence or absence of test compounds. After incubation for 24 h at 37 °C, treated RAW264.7 macrophage cells were incubated with 100 μL MTT solution (0.5 mg/mL in medium) for another 4 h at 37 °C, subsequently, the supernatants were removed and residues were dissolved using 150 μL DMSO for each well; 50 μL of cell-free supernatant was mixed with 100 μL of Griess reagent containing equal volumes of 2% (*w/v*) sulfanilamide in 5% (*w/v*) phosphoric acid and 0.2% (*w/v*) *N*-(1-naphthyl) ethylenediamine solution to measure nitrite production. The absorbance was detected at 540 nm using a microplate reader (Thermo, Waltham, MA, USA). Compared with a calibration curve prepared using NaNO_2_ standards. The experiments were performed in triplicate. quercetin was conducted as a positive control. All the compounds were prepared as stock solutions in DMSO (final solvent concentration less than 0.3% in all assays).

### 3.5. Bioactivity Evaluation

Compounds **1**–**16** were evaluated for their inhibitory effects on the NO production in LPS-stimulated RAW 264.7 macrophage cells. Quercetin was used as a positive control (IC_50_ = 7.2 ± 0.2 μM). Compounds **2**–**4**, **6**, **7**, **10**, and **11** exhibited weak inhibitory activity against NO production with IC_50_ values of 90.4 ± 3.2 μM, 74.3 ± 1.8 μM, 88.1 ± 2.2 μM, 98.4 ± 3.6 μM, 97.5 ± 2.6 μM, 97.7 ± 3.5 μM, 25.5 ± 1.2 μM, respectively.

## Figures and Tables

**Table 1:** Data of compounds **1**–**4** (500 MHz for ^1^H and 125 MHz for ^13^C, CD_3_OD, *J* in Hz).

No.	1 ^a^		2 ^a^		3 ^a^		4 ^a^	
	*δ*_H_	*δ*_C_	*δ*_H_	*δ*_C_	*δ*_H_	*δ*_C_	*δ*_H_	*δ*_C_
1		134.1		130.8		133.5		133.3
2	6.78, s	105.2	7.28, d, (1.5)	113.3	7.01, d, (1.5)	111.6	7.08, d, (1.5)	112.2
3		149.4		148.7		149.0		148.5
4		136.3		147.1		147.5		147.0
5		149.4	6.83, d, (8.0)	115.5	6.77, d, (8.5)	116.1	6.78, d, (8.0)	115.7
6	6.78, s	105.2	6.96, dd, (8.0, 1.5)	122.1	6.88, dd, (8.0, 1.5)	121.1	6.94, dd, (8.0, 1.5)	121.0
7	5.12, d, (8.0)	85.6	5.31, d, (3.0)	77.7	4.97, d, (8.5)	75.0	5.12, d, (7.0)	74.4
8	2.75, m	52.7	4.23, m	86.8	4.01, m	89.7	4.16, m	88.3
9	3.80, dd, (10.0, 4.5) 4.09, dd, (10.0, 5.5)	69.3	3.16, m	61.4	3.69, m	61.3	3.62, m	62.1
1′		133.6		135.1		140.0		133.3
2′	6.80, s	104.8	6.53, s	106.7	6.46, s	109.3	6.59, s	106.7
3′		149.3		154.3		152.0		153.9
4′		136.4		139.9		135.3		140.1
5′		149.3		154.3		152.0		153.9
6′	6.80, s	104.8	6.53, s	106.7	6.60, s	112.1	6.59, s	106.7
7′	4.98, d, (9.0)	84.2	2.67, t, (7.5)	33.4	2.56, t, (7.5)	33.0	2.69, t, (7.5)	33.4
8′	2.45, m,	51.1	1.86, m	35.4	1.80, m	35.2	1.87, m	35.4
9′	3.72, (dd,12.0, 5.0) 4.36, overlapped	64.8	3.61, t, (6.4)	62.8	3.56, t, (6.5)	62.2	3.81, dd, (11.0, 2.5) 3.93, dd, (11.0, 4.0)	69.2
Glu-1′′	4.36, d, (8.0)	104.6	4.23, d, (7.5)	101.0	4.93, d, (7.5)	103.0	4.32, d, (8.0)	104.5
Glu-2′′	3.26, overlapped	75.2	3.45, overlapped	75.2	3.48, overlapped	75.1	3.21, overlapped	75.4
Glu-3′′	3.40, overlapped	78.1	3.45, overlapped	77.8	3.41, overlapped	78.0	3.25, overlapped	77.9
Glu-4′′	3.33, overlapped	71.6	3.32, overlapped	71.9	3.40, overlapped	71.4	3.26, overlapped	71.8
Glu-5′′	3.36, overlapped	78.2	3.45, overlapped	78.1	3.47, overlapped	78.3	3.28, overlapped	78.0
Glu-6′′	4.32, overlapped 4.36, overlapped	62.8	3.87, overlapped 3.92, overlapped	62.2	3.68, overlapped 3.89, overlapped	62.5	3.68, overlapped 3.89, overlapped	62.9
COCH_3_		172.8						
COCH_3_	1.95 (3H, s)	20.7						
3-OCH_3_	3.93 (3H, s)	56.9	3.89 (3H, s)	56.4	3.86, (3H, s)	56.4	3.89, (3H, s)	56.5
5-OCH_3_	3.93 (3H, s)	56.9						
3′-OCH_3_	3.93 (3H, s)	56.9	3.74 (3H, s)	56.4			3.89, (3H, s)	56.6
5′-OCH_3_	3.93 (3H, s)	56.9	3.74 (3H, s)	56.4			3.89, (3H, s)	56.6

^a^ Assignments were carried out based on HSQC and HMBC experiments.

## Supplementary Materials

The following materials are available online: HRESIMS and NMR spectra data of compounds **1–4** as supporting information.
