# Supplementary material for: Lignan Glycosides from Urena lobata
Source: Molecules. 2019 Aug 6;24(15):2850. doi: 10.3390/molecules24152850 (PMC6696325; doi:10.3390/molecules24152850)
Supplement: Supplementary file 1 [file molecules-24-02850-s001.zip › molecules-556950-SI.pdf]

Supporting Information

Lignan Glycosides from *Urena lobata*

Yuan Luo[1], Cong Su[1], Ning Ding[1], Bowen Qi[1], Fangfang Jia[1], Xiping Xu[1], Xiao Liu[1], Juan Wang[1], Xiaohui Wang[1], Pengfei Tu[1] and Shepo Shi[1, 2, *]

[1] Modern Research Center for Traditional Chinese Medicine, Beijing University of Chinese Medicine, Beijing 100029, People's Republic of China

[2] Beijing Key Lab for Quality Evaluation of Chinese Meteria Medica, Beijing University of Chinese Medicine, Beijing 100029, China.

* Correspondence: shishepo@163.com (S.-P.S.); Tel./Fax.: +86-010-6428-6350

The List of Contents

**No.**	Content
**S1**	HRESIMS spectrum of compound **1**
**S2**	^{1}H NMR spectrum of compound **1**
**S3**	^{13}C NMR spectrum of compound **1**
**S4**	^{1}H-^{1}H COSY spectrum of compound **1**
**S5**	gHSQC spectrum of compound **1**
**S6**	gHMBC spectrum of compound **1**
**S7**	NOESY spectrum of compound **1**
**S8**	CD spectrum of compound **1**
**S9**	HRESIMS spectrum of compound **2**
**S10**	^{1}H NMR spectrum of compound **2**
**S11**	^{13}C NMR spectrum of compound **2**
**S12**	^{1}H-^{1}H COSY spectrum of compound **2**
**S13**	gHSQC spectrum of compound **2**
**S14**	gHMBC spectrum of compound **2**
**S15**	NOESY spectrum of compound **2**
**S16**	CD spectrum of compound **2**
**S17**	HRESIMS spectrum of compound **3**
**S18**	^{1}H NMR spectrum of compound **3**
**S19**	^{13}C NMR spectrum of compound **3**
**S20**	^{1}H-^{1}H COSY spectrum of compound **3**
**S21**	gHSQC spectrum of compound **3**
**S22**	gHMBC spectrum of compound **3**
**S23**	NOESY spectrum of compound **3**
**S24**	CD spectrum of compound **3**
**S25**	HRESIMS spectrum of compound **4**
**S26**	^{1}H NMR spectrum of compound **4**
**S27**	^{13}C NMR spectrum of compound **4**
**S28**	^{1}H-^{1}H COSY spectrum of compound **4**
**S29**	gHSQC spectrum of compound **4**
**S30**	gHMBC spectrum of compound **4**
**S31**	NOESY spectrum of compound **4**
**S32**	CD spectrum of compound **4**

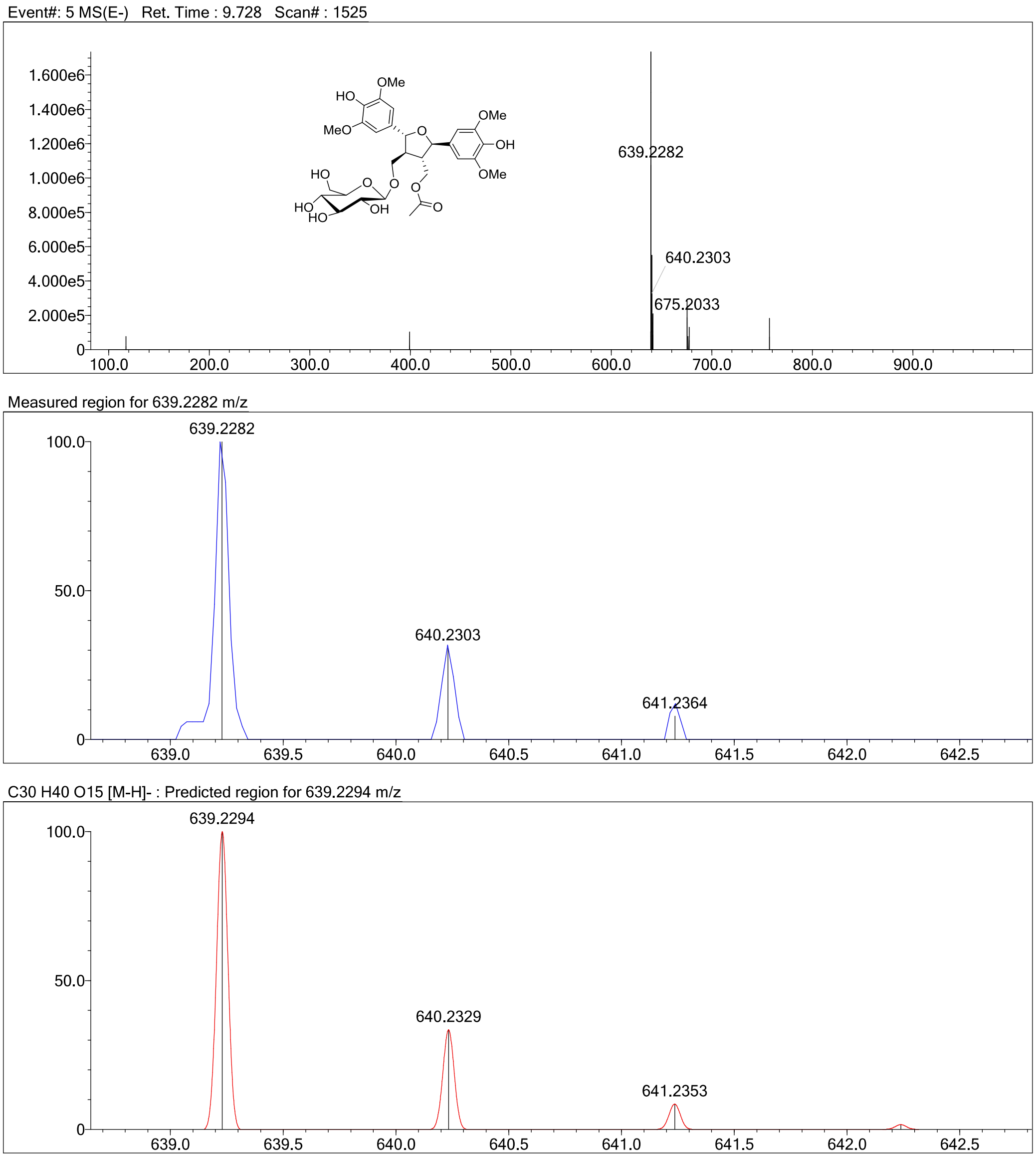

Rank	Score	Formula (M)	Ion	Meas. m/z	Pred. m/z	Df. (mDa)	Df. (ppm)	Iso	DBE
1	77.91	C30 H40 O15	[M-H]-	639.2282	639.2294	-1.2	-1.88	79.67	11.0

S1 HRESIMS spectrum of compound **1**

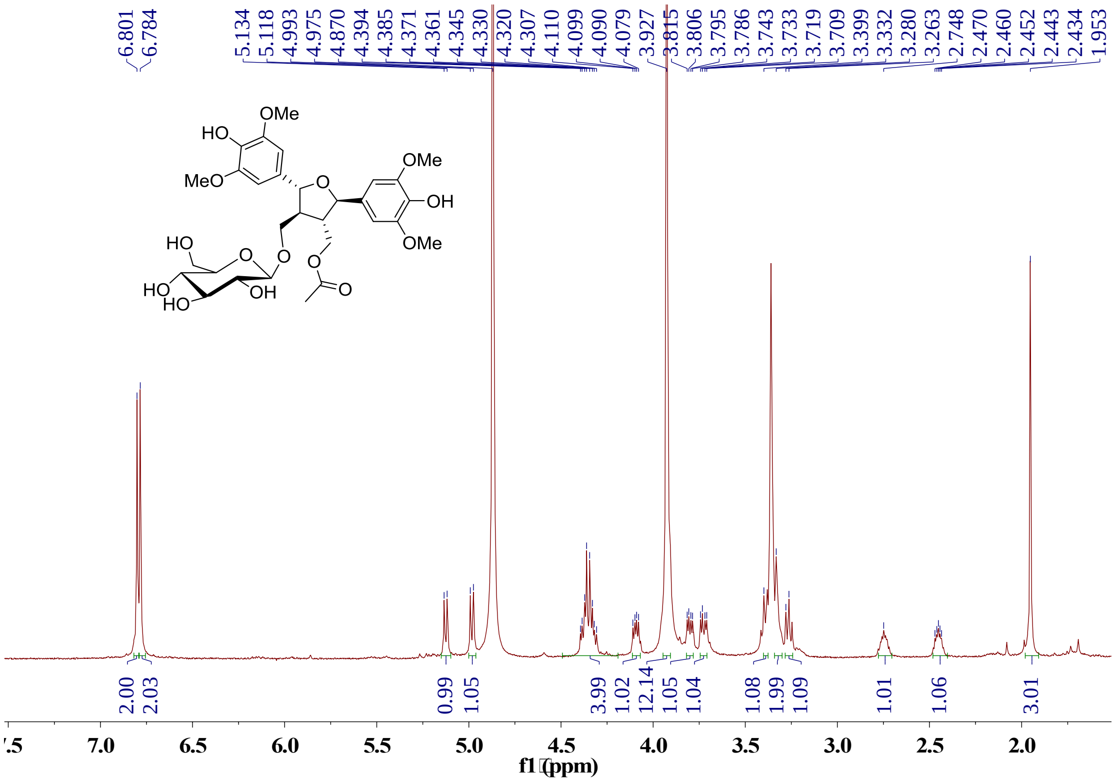

S2 ^{1}H NMR spectrum of compound **1**

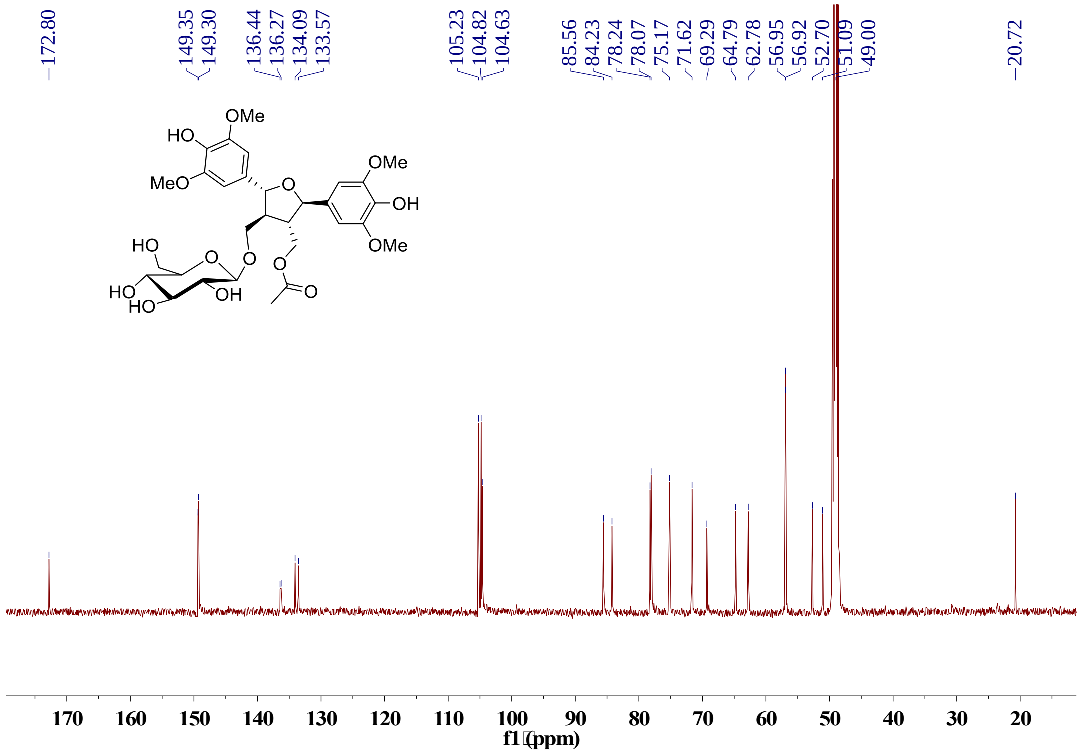

S3 ^{13}C NMR spectrum of compound **1**

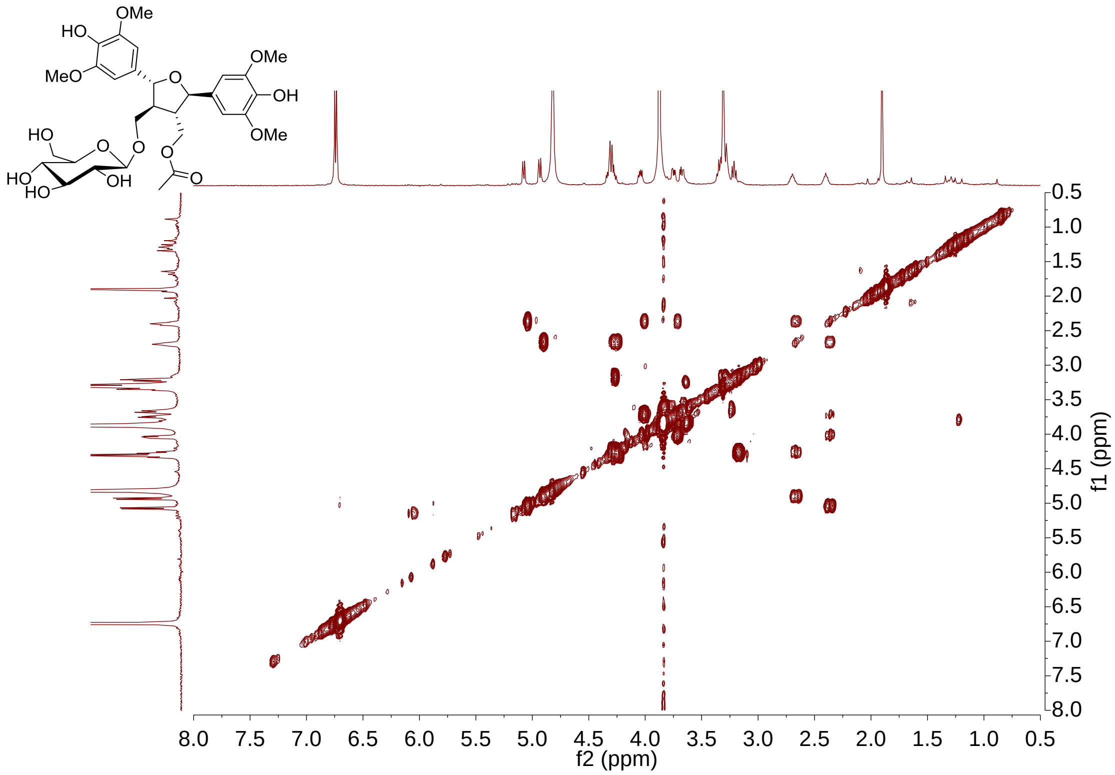

S4 ^{1}H-^{1}H COSY spectrum of compound **1**

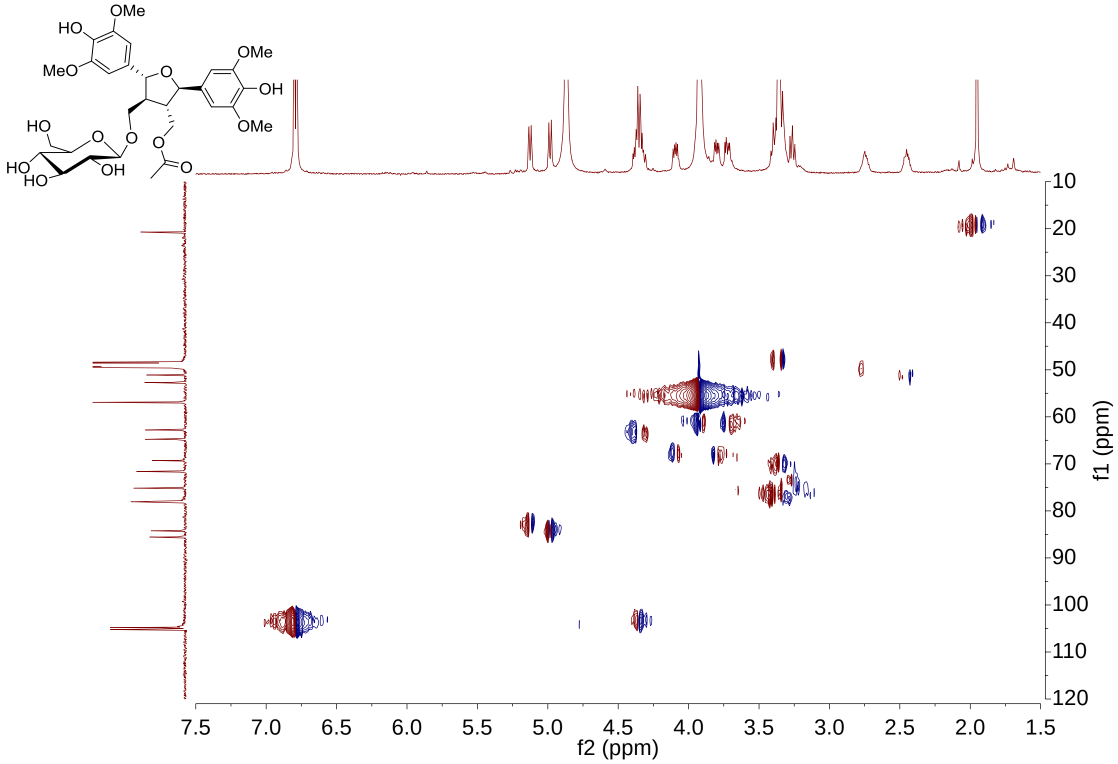

S5 gHSQC spectrum of compound **1**

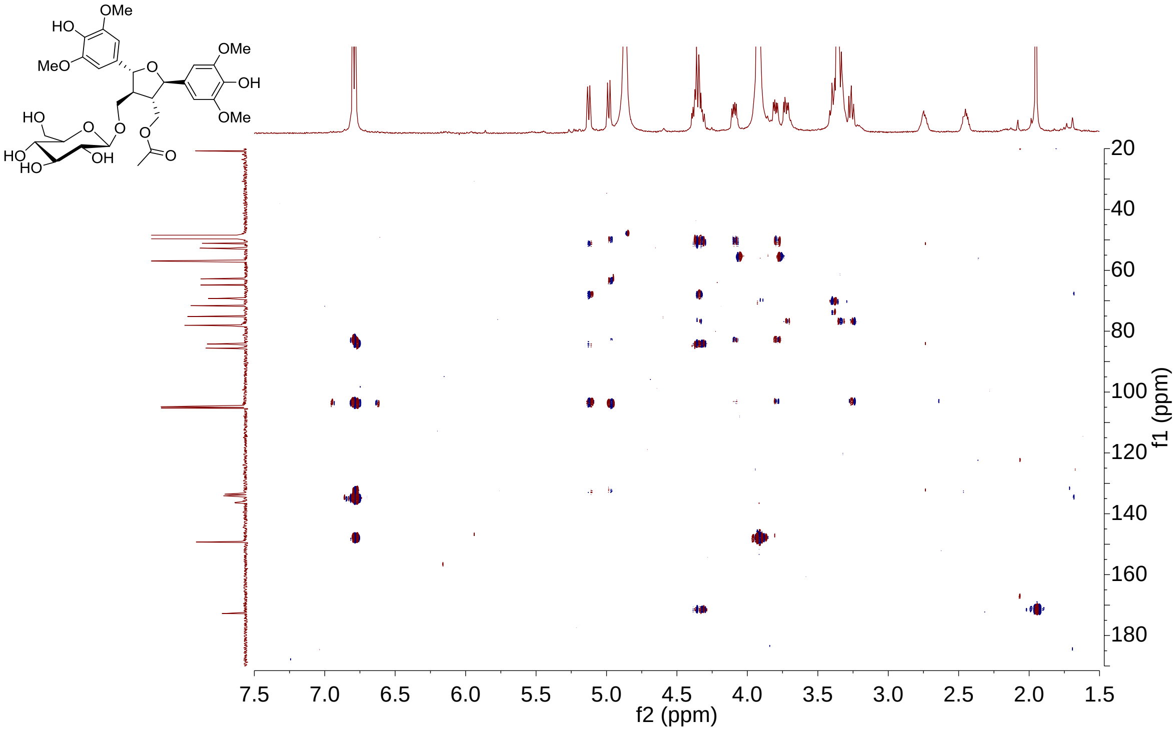

S6 gHMBC spectrum of compound **1**

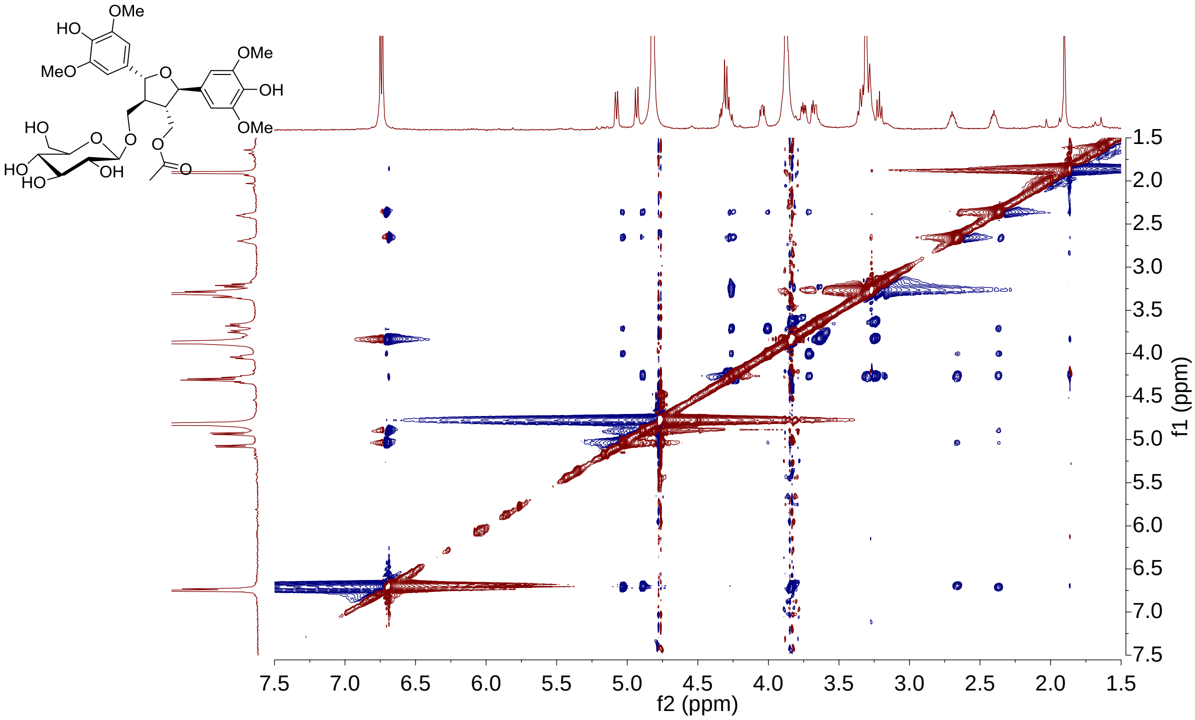

S7 NOESY spectrum of compound **1**

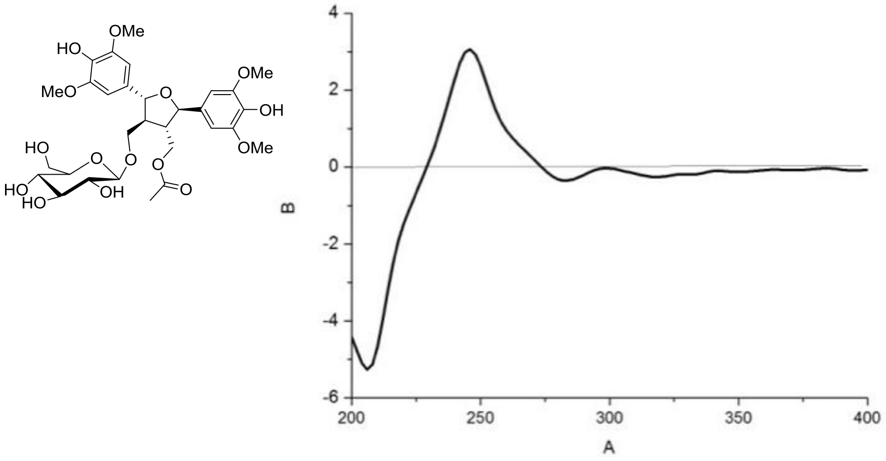

S8 CD spectrum of compound **1**

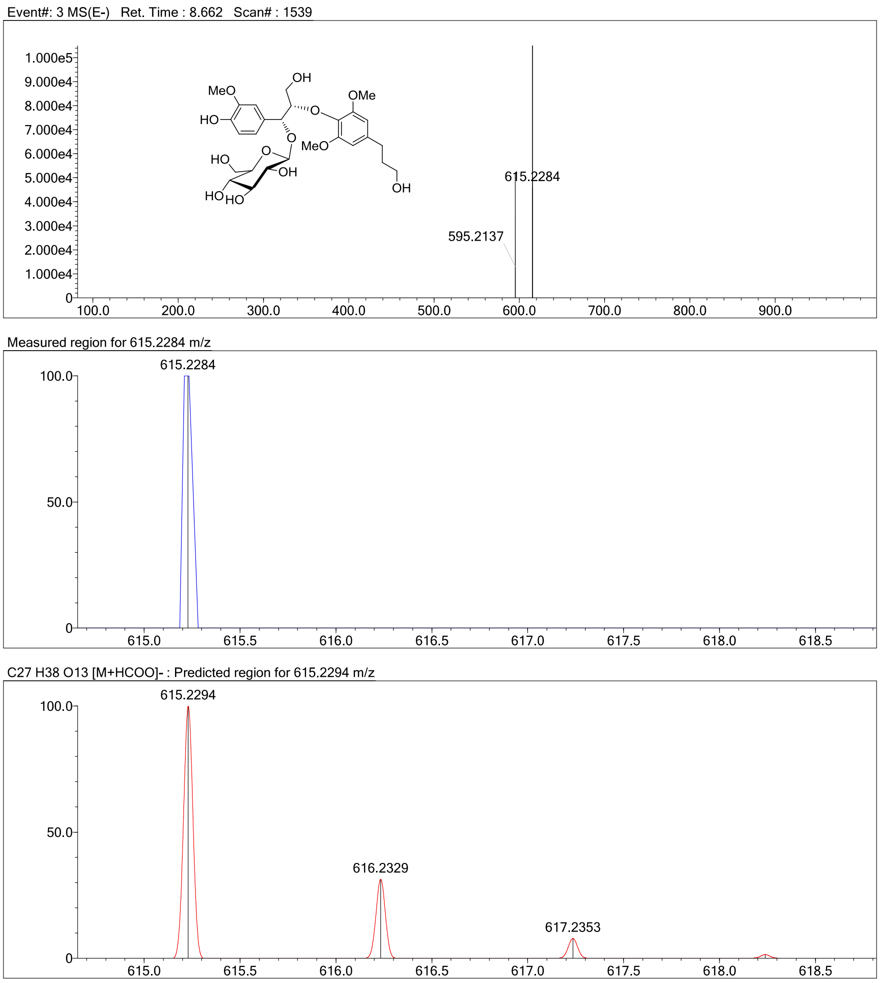

Rank	Score	Formula (M)	Ion	Meas. m/z	Pred. m/z	Df. (mDa)	Df. (ppm)	Iso	DBE
2	0.00	C27 H38 O13	[M+HCOO]-	615.2284	615.2294	-1.0	-1.63	0.00	9.0

S9 HRESIMS spectrum of compound **2**

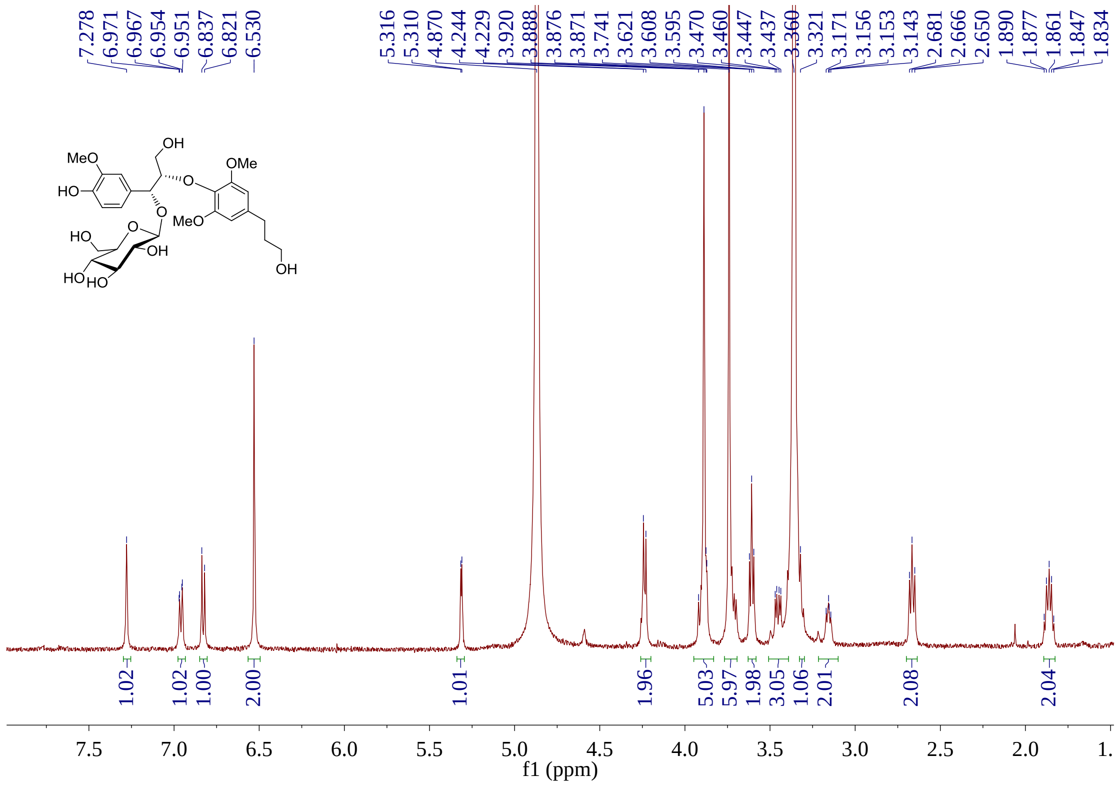

S10 ^{1}H NMR spectrum of compound **2**

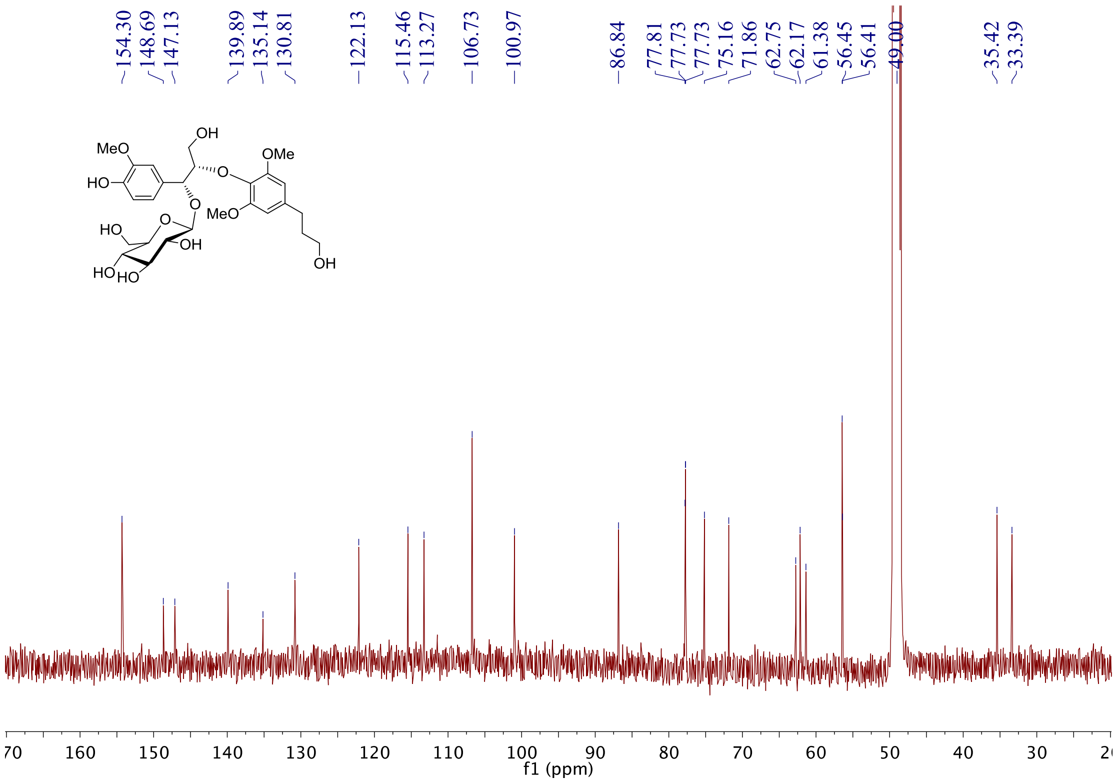

S11 ^{13}C NMR spectrum of compound **2**

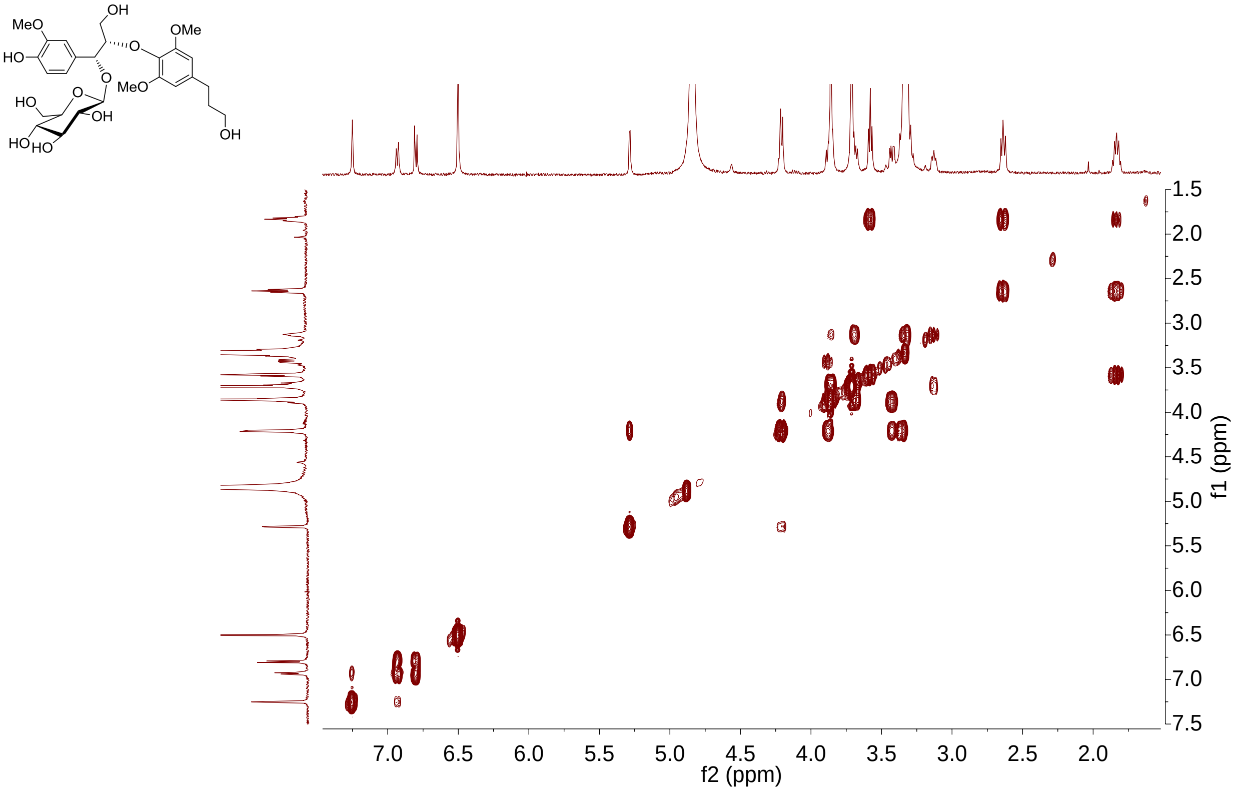

S12 ^{1}H-^{1}H COSY spectrum of compound **2**

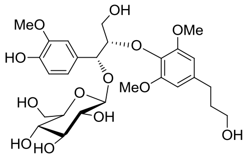

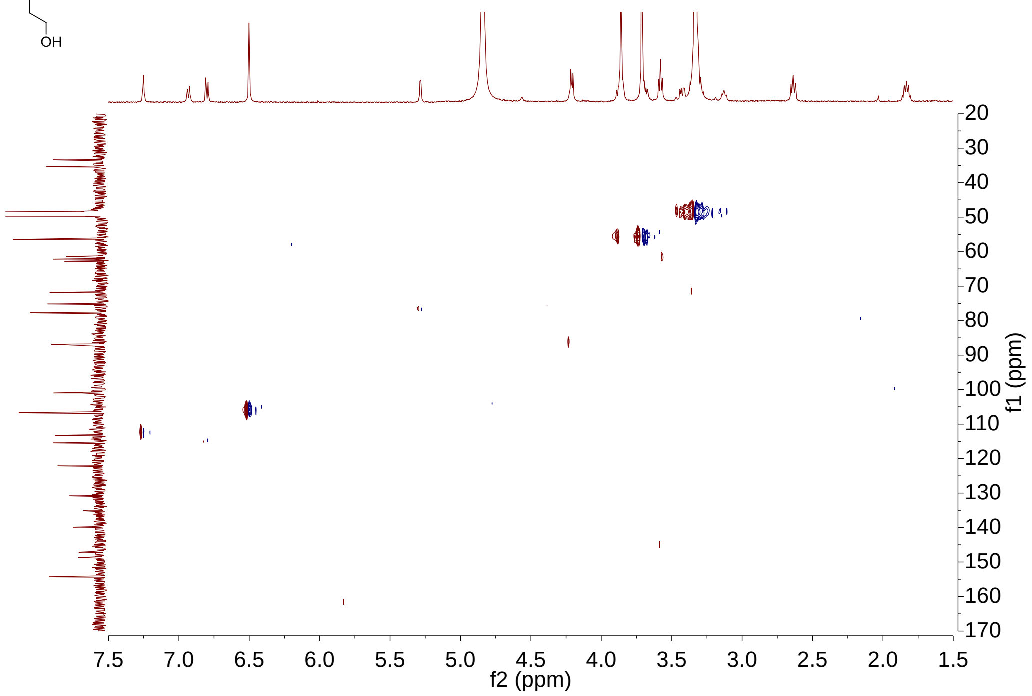

S13 gHSQC spectrum of compound **2**

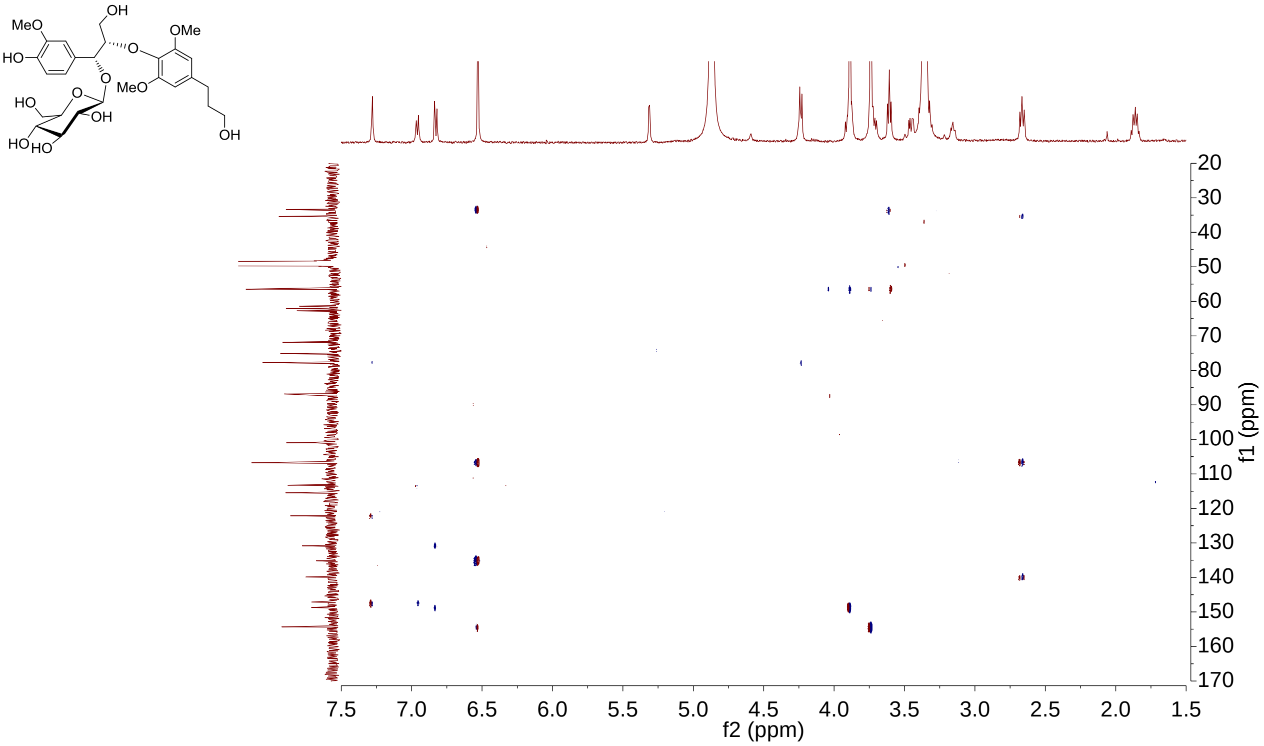

S14 gHMBC spectrum of compound **2**

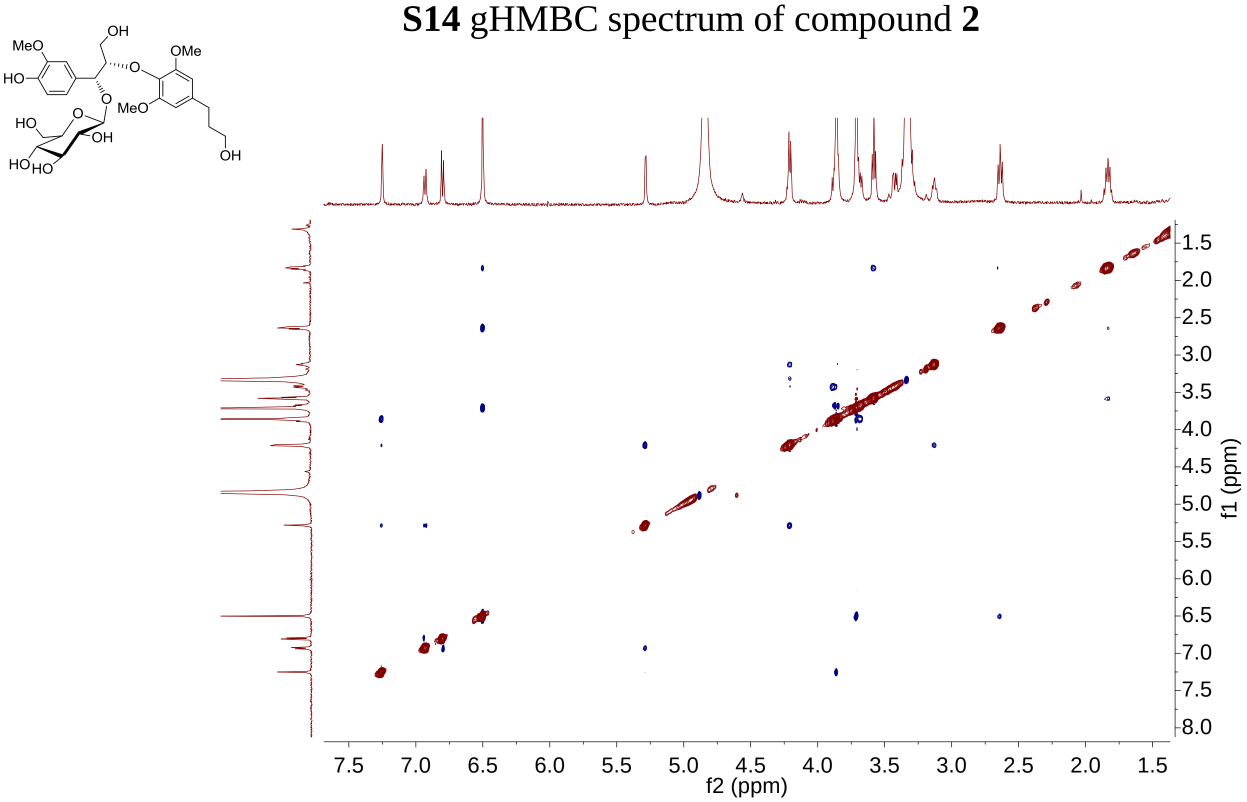

S15 NOESY spectrum of compound **2**

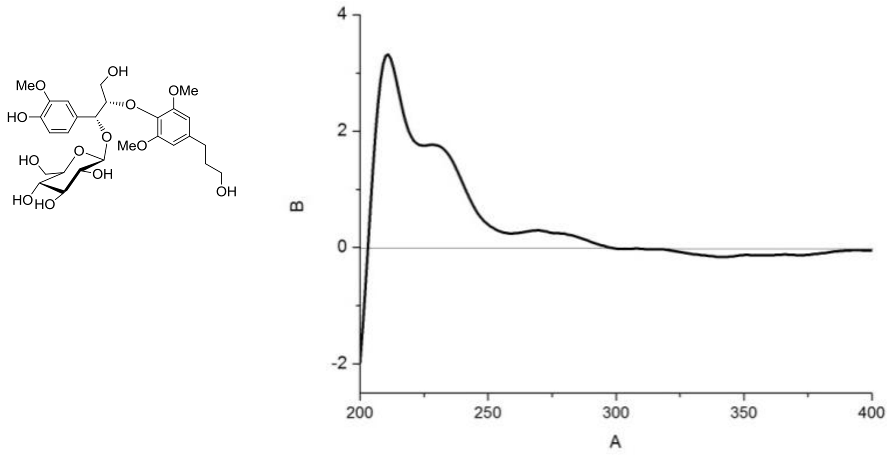

S16 CD spectrum of compound **2**

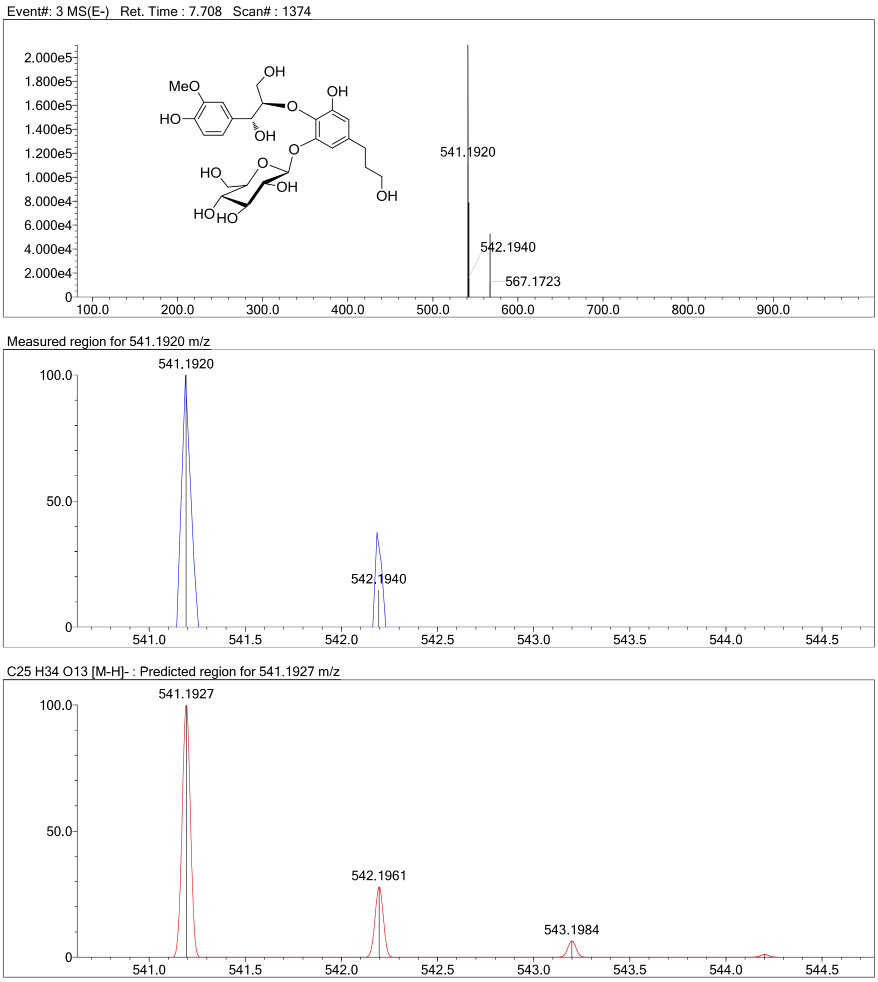

Rank	Score	Formula (M)	Ion	Meas. m/z	Pred. m/z	Df. (mDa)	Df. (ppm)	Iso	DBE
1	0.00	C25 H34 O13	[M-H]-	541.1920	541.1927	-0.7	-1.29	0.00	9.0

S17 HRESIMS spectrum of compound **3**

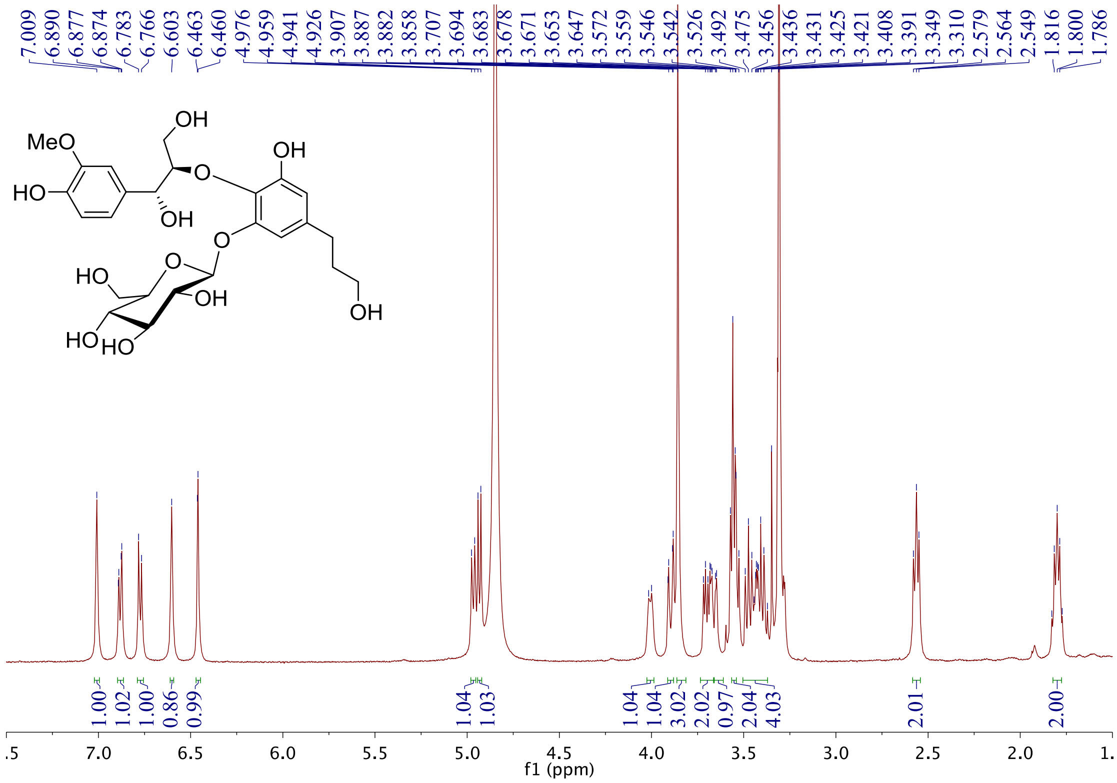

S18 ^{1}H NMR spectrum of compound **3**

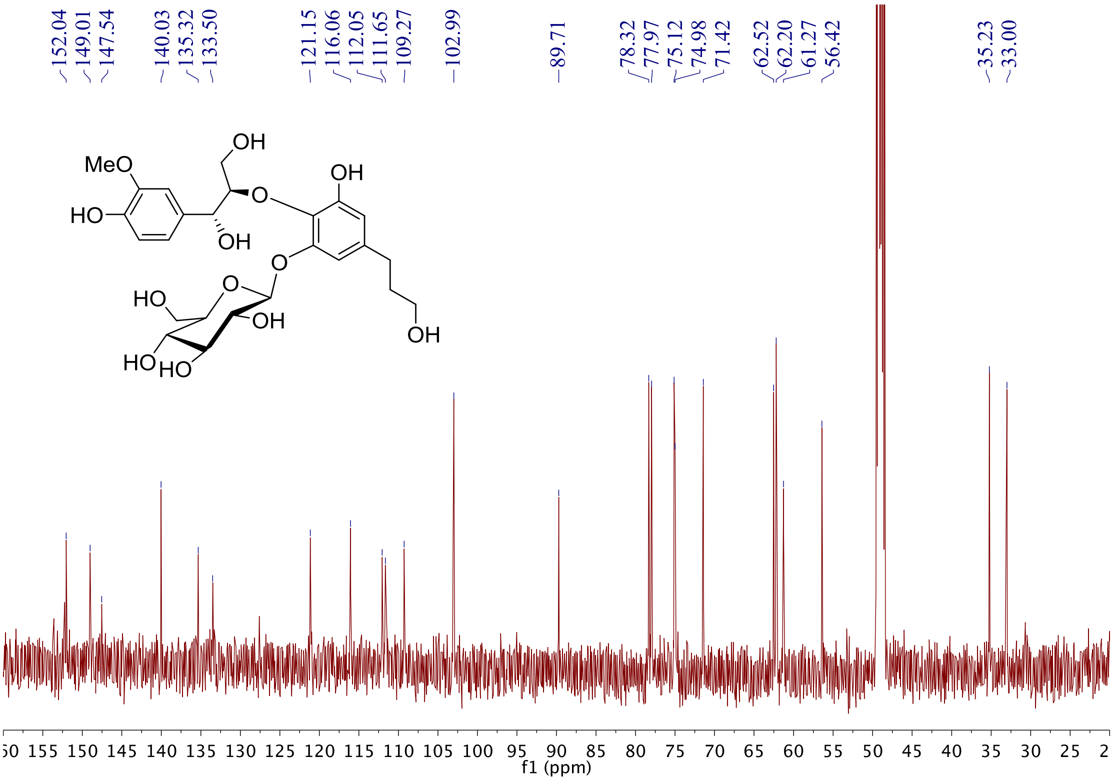

S19 ^{13}C NMR spectrum of compound **3**

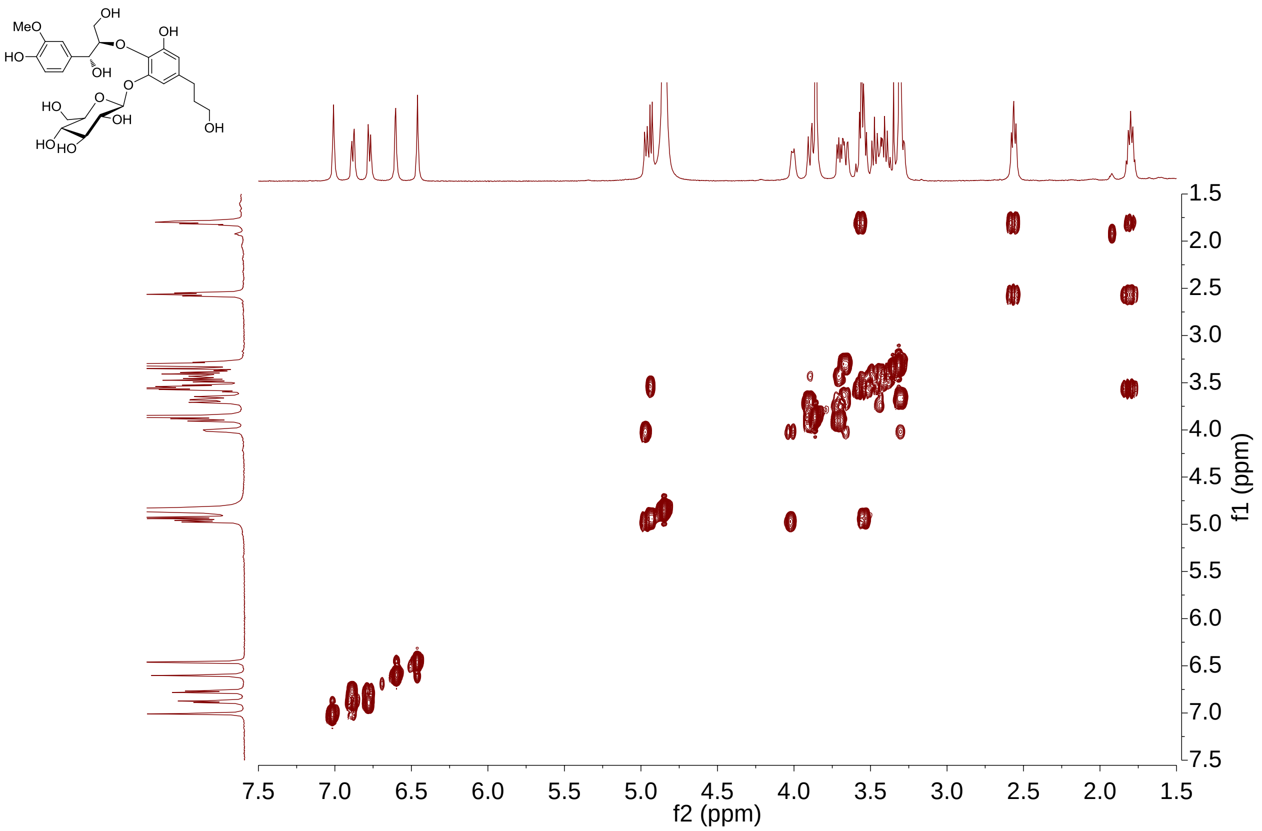

S20 ^{1}H-^{1}H COSY spectrum of compound **3**

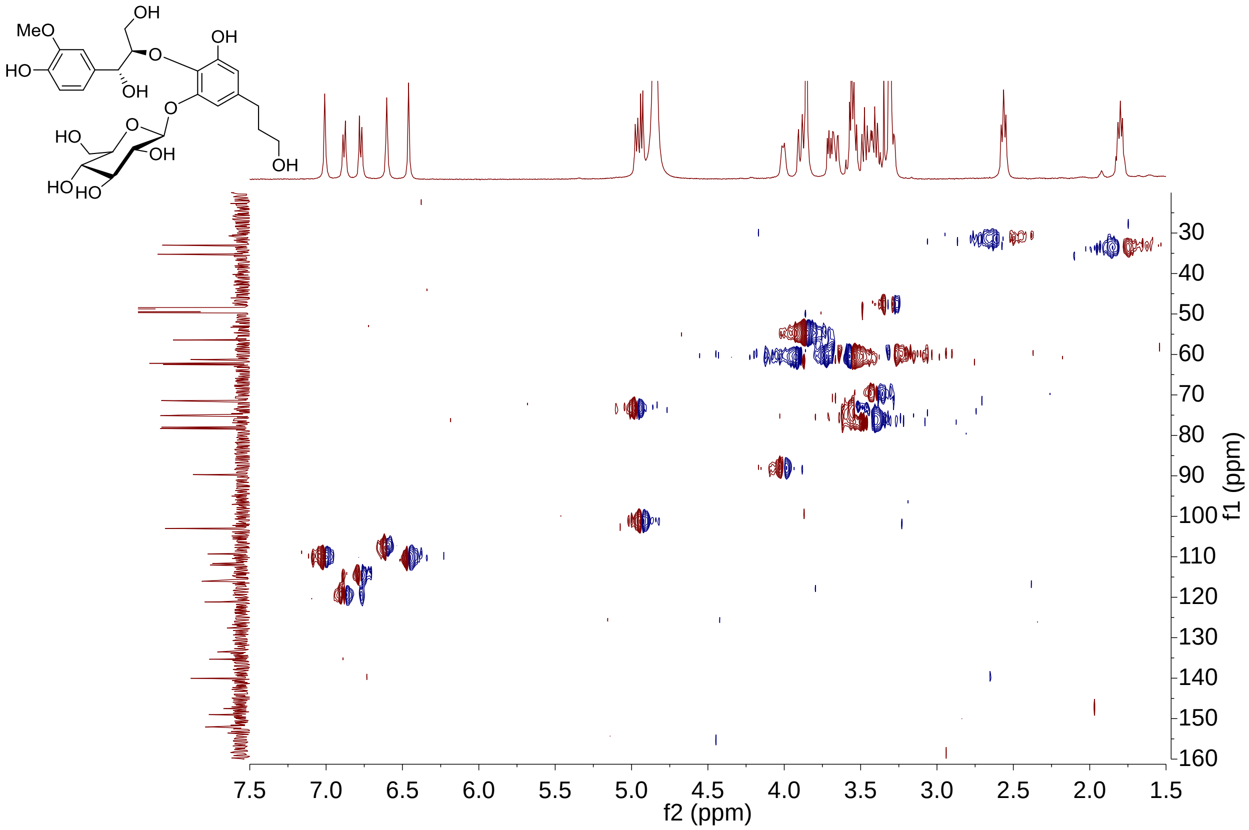

S21 gHSQC spectrum of compound **3**

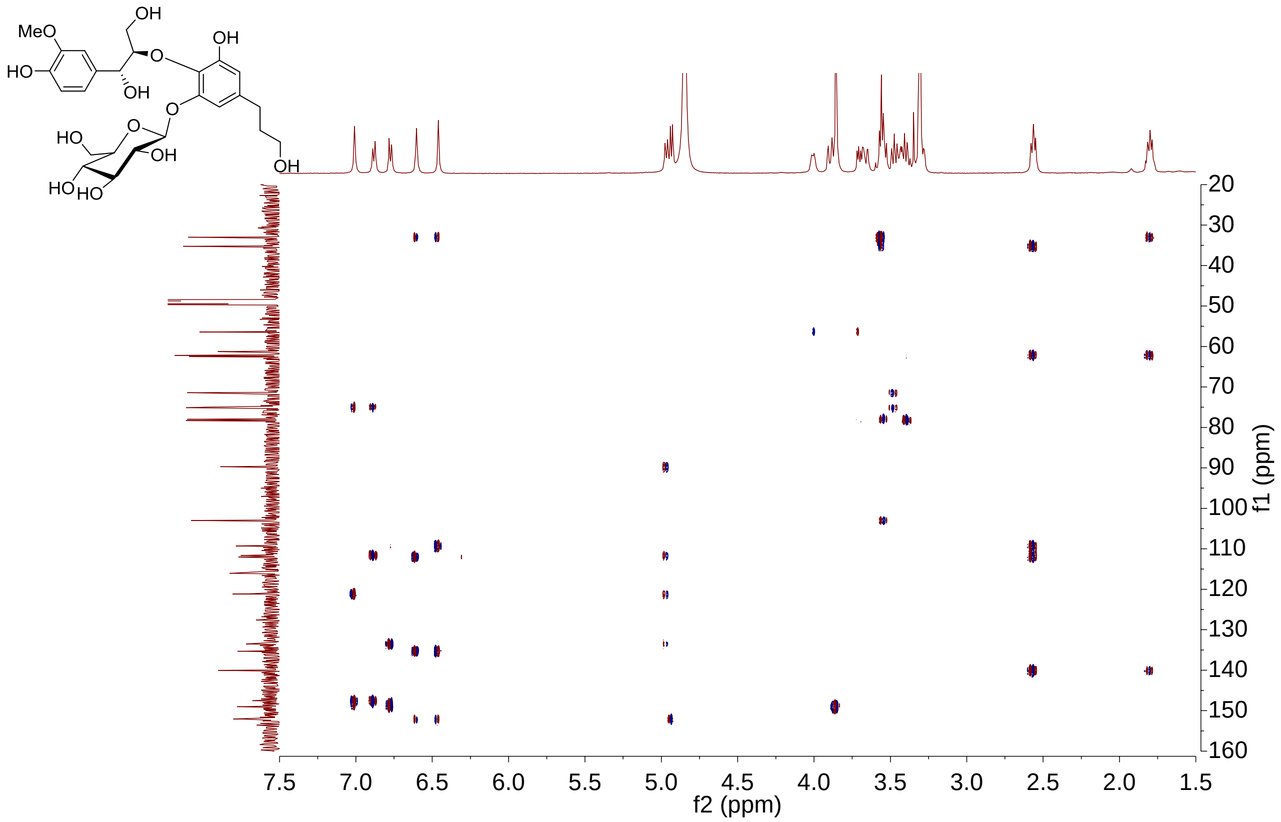

S22 gHMBC spectrum of compound **3**

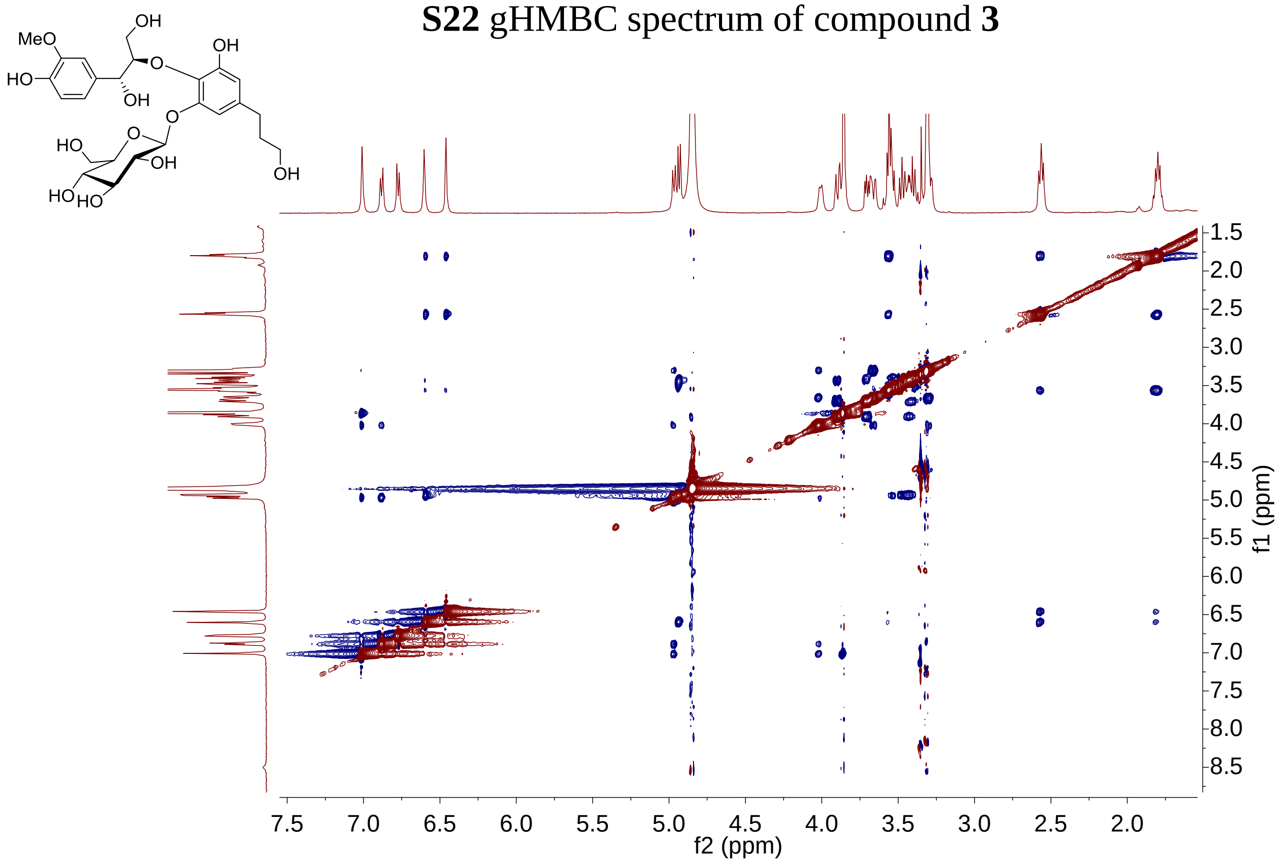

S23 NOESY spectrum of compound **3**

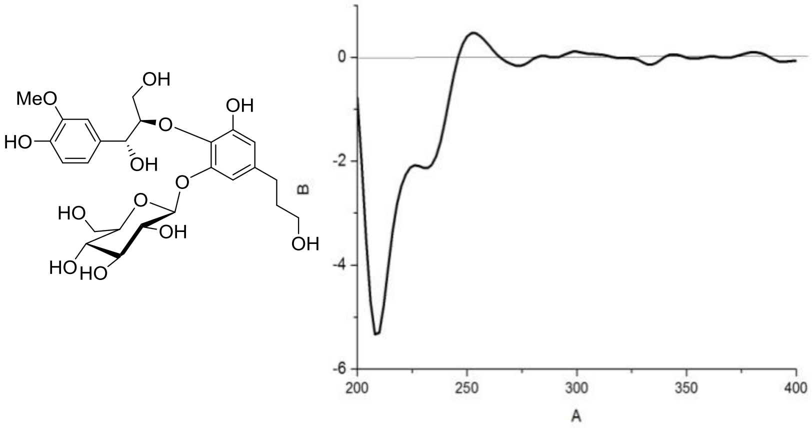

S24 CD spectrum of compound **3**

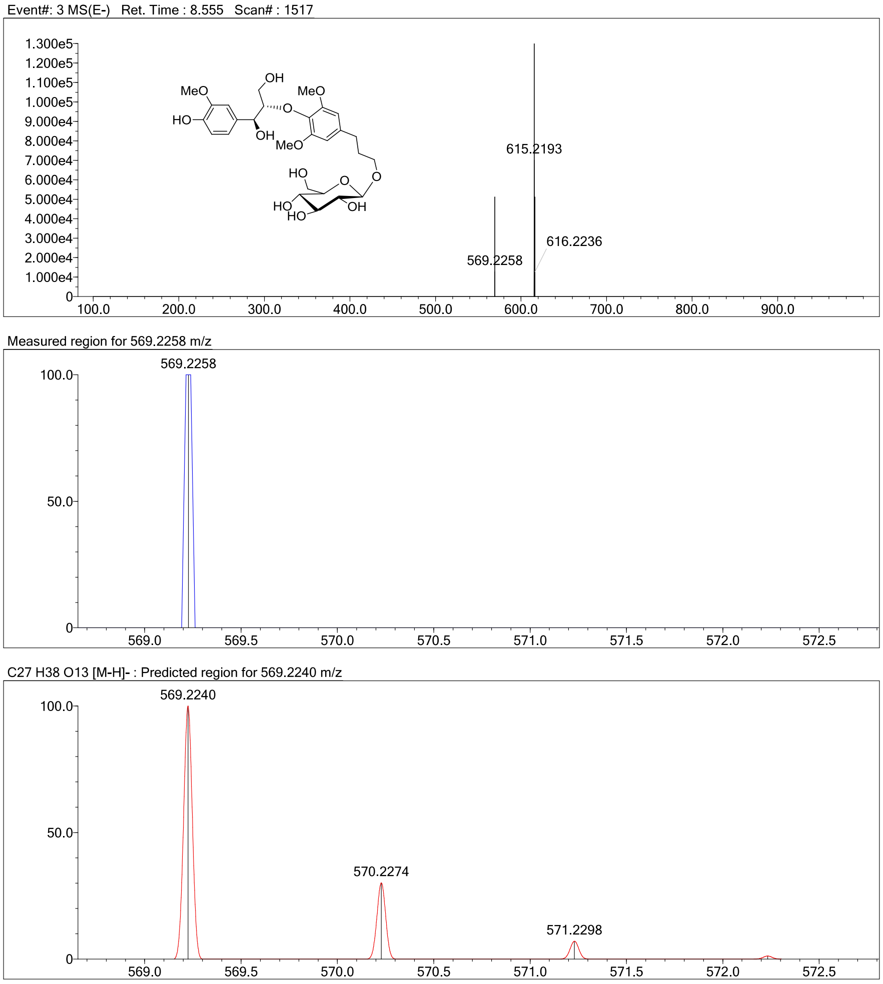

Rank	Score	Formula (M)	Ion	Meas. m/z	Pred. m/z	Df. (mDa)	Df. (ppm)	Iso	DBE
2	0.00	C27 H38 O13	[M-H]-	569.2258	569.2240	1.8	3.16	0.00	9.0

S25 HRESIMS spectrum of compound **4**

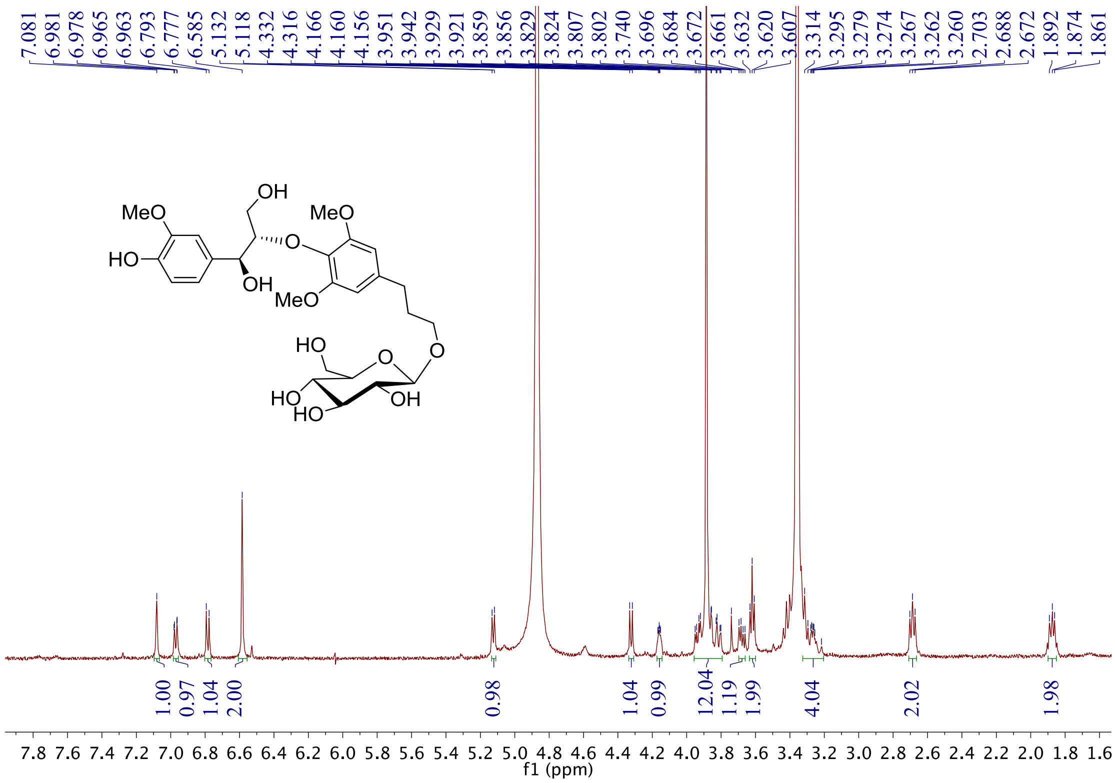

S26 ^{1}H NMR spectrum of compound **4**

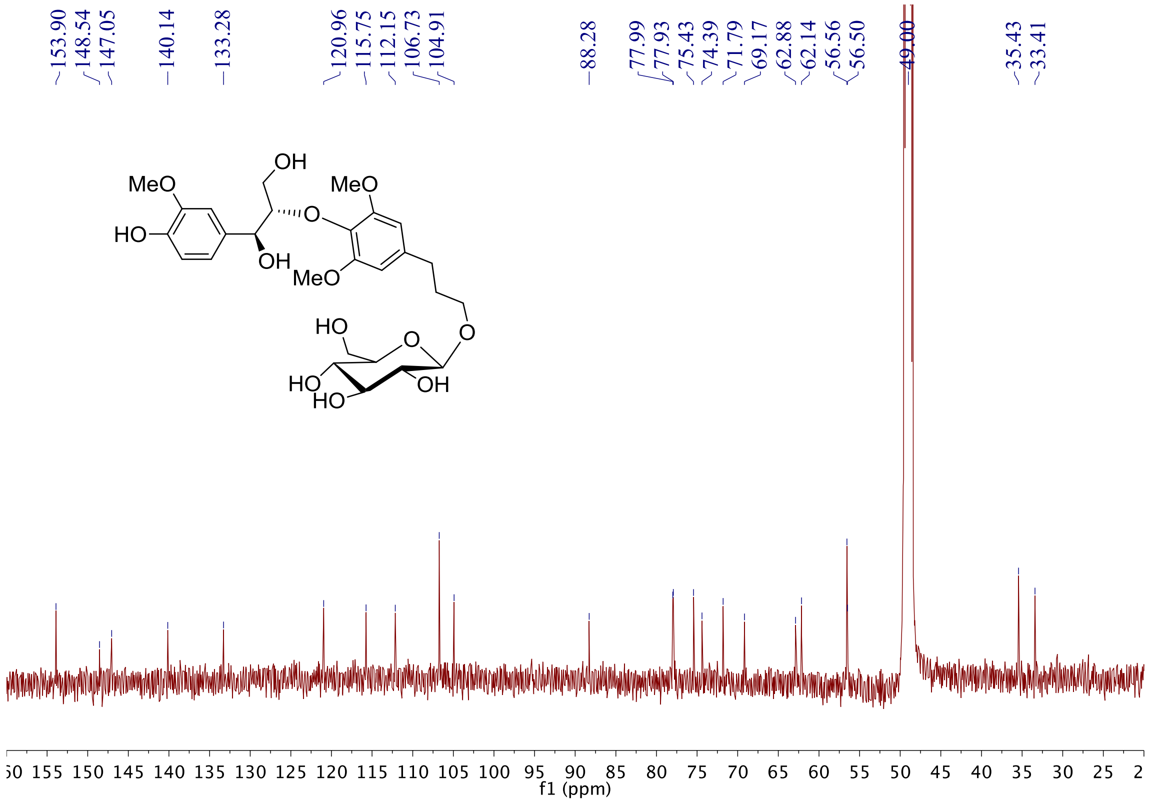

S27 ^{13}C NMR spectrum of compound **4**

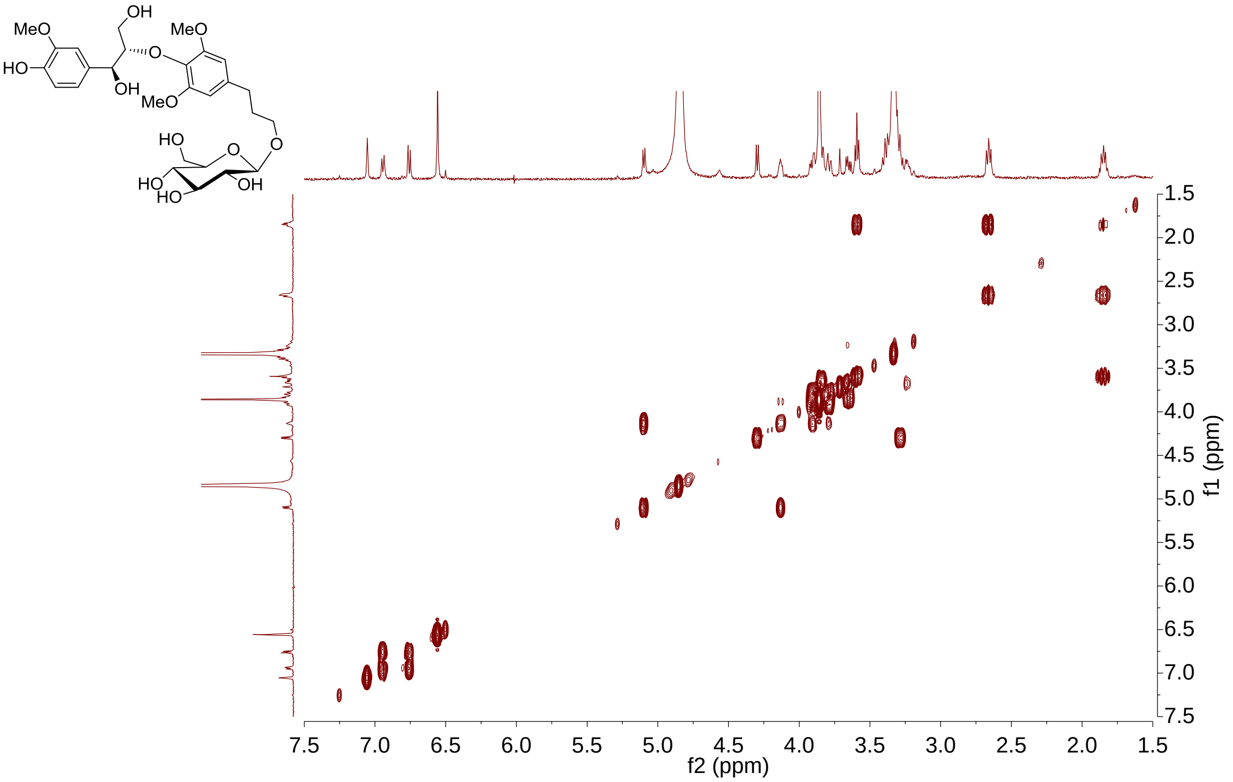

S28 ^{1}H-^{1}H COSY spectrum of compound **4**

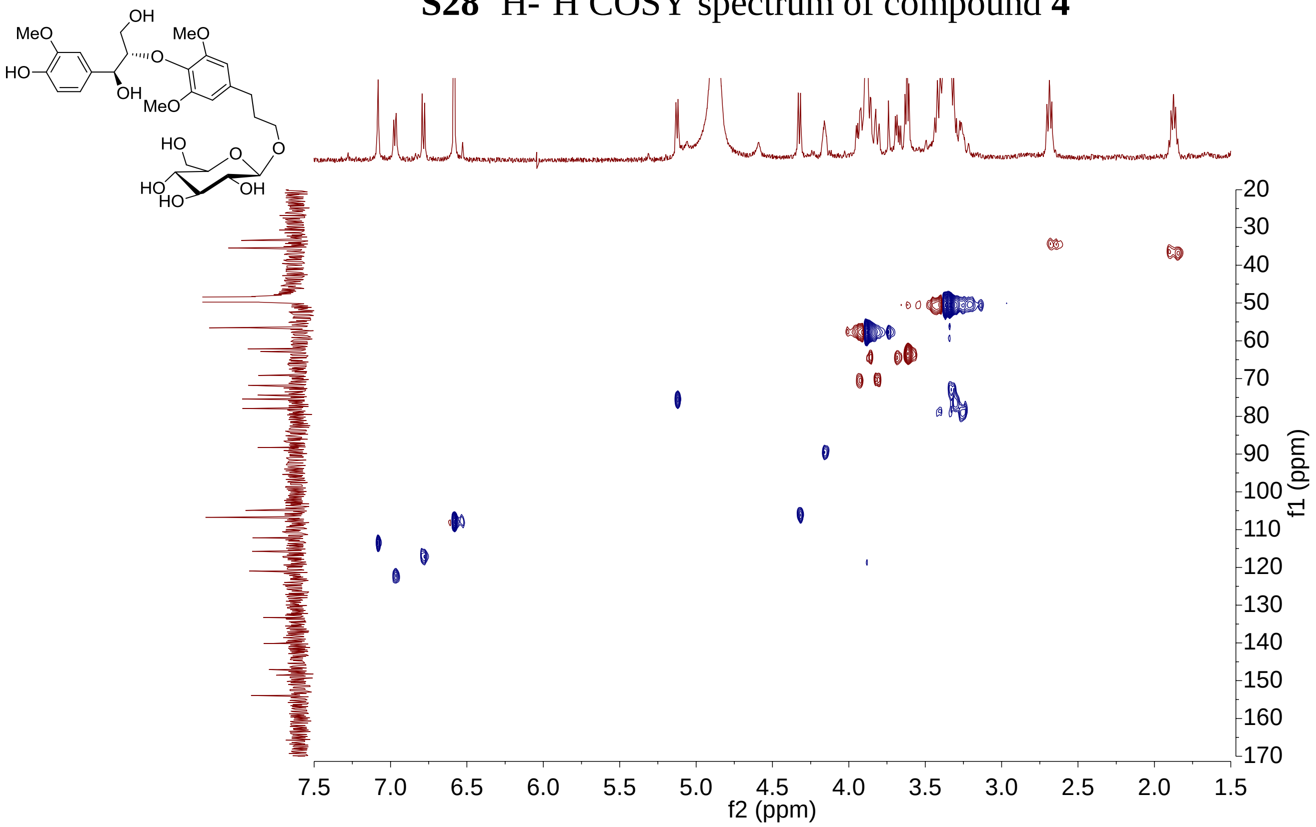

S29 gHSQC spectrum of compound **4**

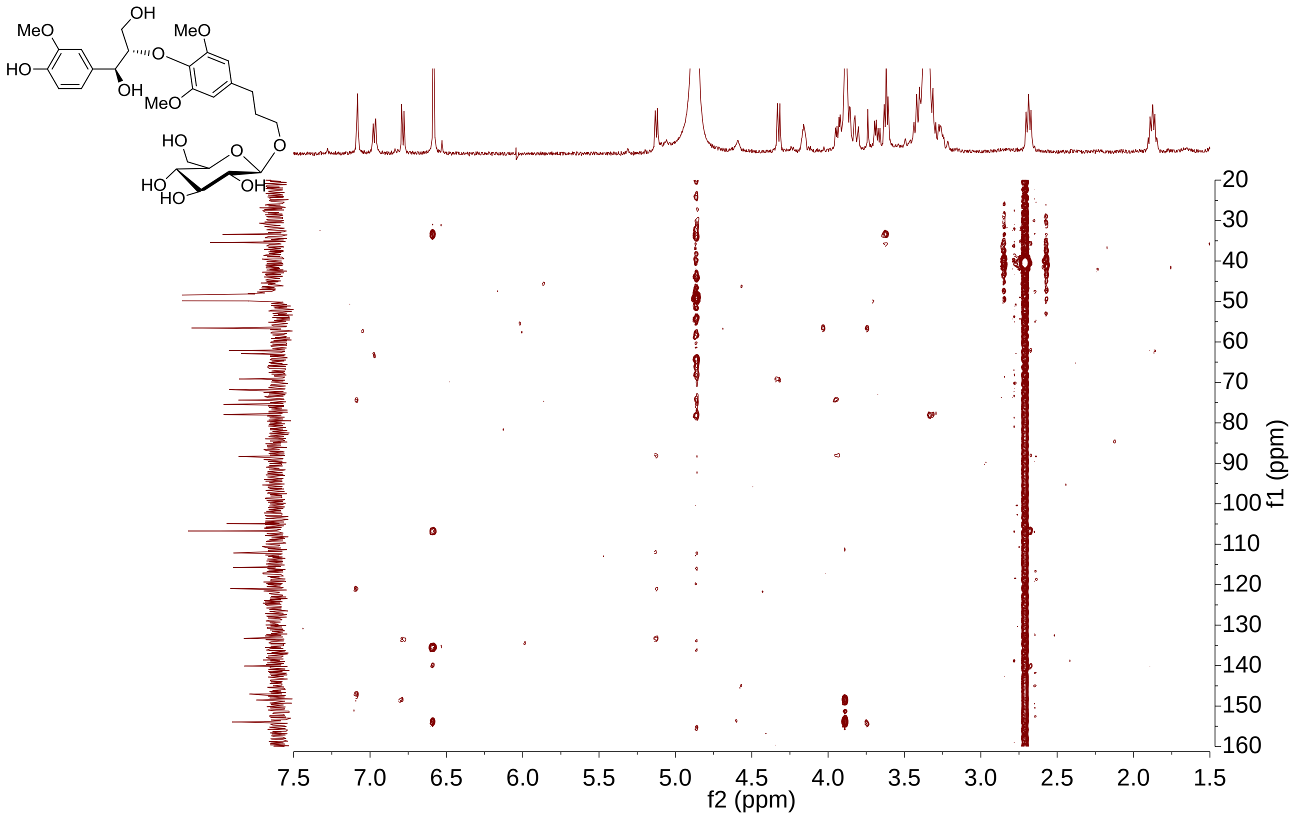

S30 gHMBC spectrum of compound **4**

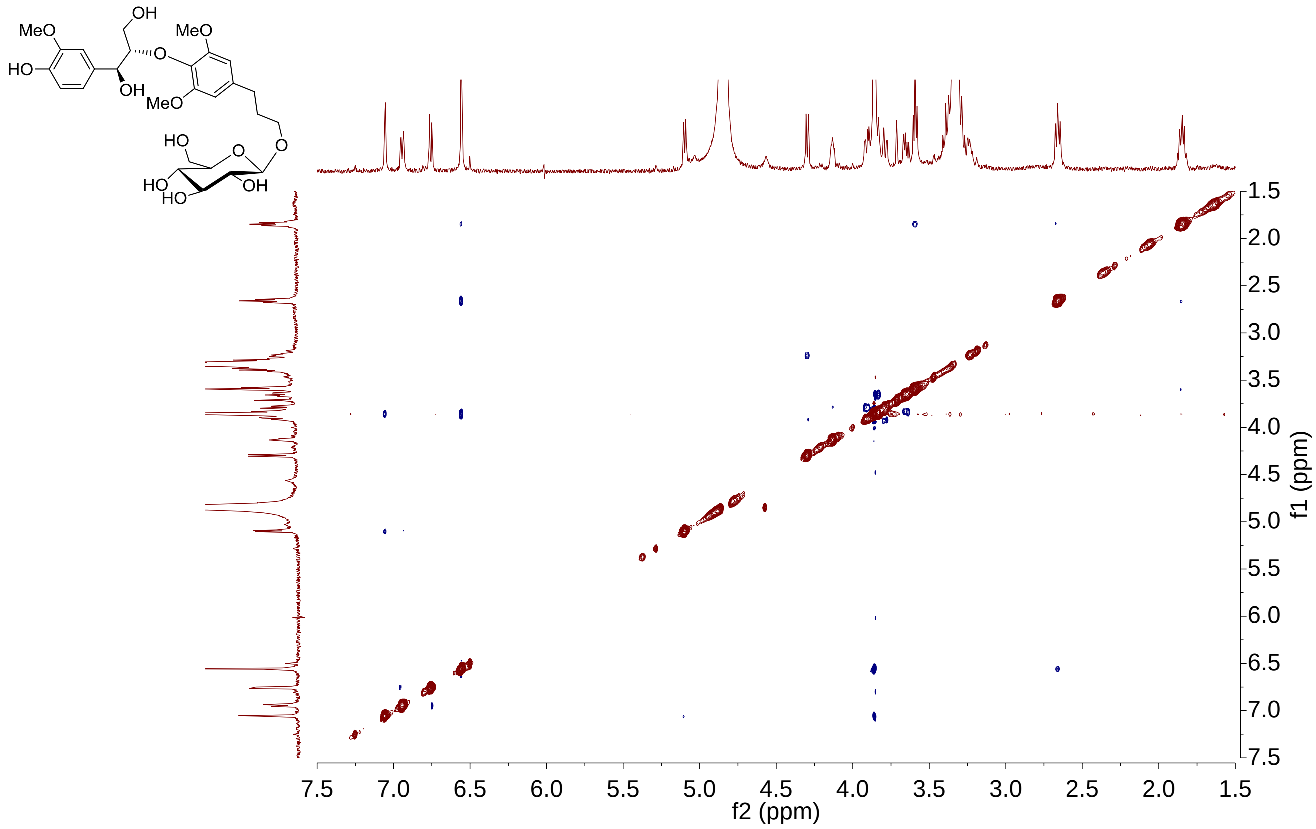

S31 NOESY spectrum of compound **4**

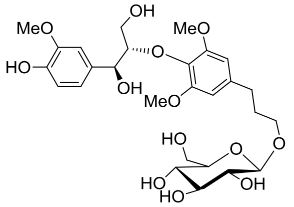

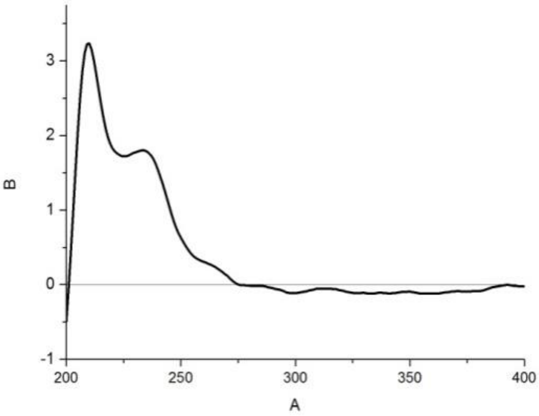

S32 CD spectrum of compound **4**
